# LncRNA–miRNA–mRNA Networks of Gastrointestinal Cancers Representing Common and Specific LncRNAs and mRNAs

**DOI:** 10.3389/fgene.2021.791919

**Published:** 2022-01-24

**Authors:** Hassan Dastsooz, Ahad Alizadeh, Parham Habibzadeh, Ali Nariman, Asieh Hosseini, Yaser Mansoori, Hamed Haghi-Aminjan

**Affiliations:** ^1^ Department of Life Sciences and Systems Biology, University of Turin, Turin, Italy; ^2^ Candiolo, C/o IRCCS, IIGM-Italian Institute for Genomic Medicine, Turin, Italy; ^3^ Candiolo Cancer (IT), FPO-IRCCS, Candiolo Cancer Institute, Turin, Italy; ^4^ Medical Microbiology Research Center, Qazvin University of Medical Sciences, Qazvin, Iran; ^5^ Research Center for Health Sciences, Institute of Health, Shiraz University of Medical Sciences, Shiraz, Iran; ^6^ Genetics and Molecular Biology Department, Isfahan University of Medical Sciences, Isfahan, Iran; ^7^ Razi Drug Research Center, Iran University of Medical Sciences, Tehran, Iran; ^8^ Department of Medical Genetics, Fasa University of Medical Sciences, Fasa, Iran; ^9^ Noncommunicable Diseases Research Center, Fasa University of Medical Sciences, Fasa, Iran; ^10^ Pharmaceutical Sciences Research Center, Ardabil University of Medical Sciences, Ardabil, Iran

**Keywords:** tumor biomarkers, gastrointestinal cancers, long-non-coding RNA, The Cancer Genome Atlas (TCGA), competitive endogenous RNA (ceRNA)

## Abstract

Gastrointestinal (GI) cancers are responsible for approximately half of cancer-related deaths, highlighting the need for the identification of distinct and common features in their clinicopathological characteristics. Long ncRNA (lncRNAs), which are involved in competitive endogenous RNA (ceRNA) networks with critical roles in biological processes, constitute a substantial number of non-coding RNAs. Therefore, our study aimed to investigate the similarities and differences in the ceRNA networks of The Cancer Genome Atlas (TCGA)-GI cancers. We performed a comprehensive bioinformatics analysis of ceRNA networks for TCGA-GI cancers in terms of the deferential mRNA, lncRNA, and miRNA expression levels, ceRNA networks, overall survival analysis, correlation analysis, pathological cancer stages, and gene set enrichment analysis. Our study revealed several common and distinct mRNAs and lncRNAs with prognostic values in these networks. It was specifically noteworthy that *MAGI2-AS3* lncRNA was found to be shared in almost all GI cancers. Moreover, the most common shared mRNAs between GI cancers were *MEIS1, PPP1R3C, ADAMTSL3, RIPOR2,* and *MYLK.* For each cancer ceRNA network, we found that the expression level of a number of lncRNAs and mRNAs was specific. Furthermore, our study provided compelling evidence that several genes, most notably *KDELC1*, can act as novel proto-oncogenes in cancers. This, in turn, can highlight their role as new prognostic and therapeutic targets. Moreover, we found cell cycle and extracellular matrix structural constituent as the top shared KEGG and molecular function, respectively, among GI cancers. Our study revealed several known lncRNAs and known and unknown mRNAs in GI cancers with diagnostic and prognostic values.

## 1 Introduction

The gastrointestinal (GI) system consists of the substantial cellular mass in the human body, and its cancers are among the most common malignancies in different populations, accounting for 35% of the total deaths due to cancers (Arnold et al., 2020). Over the past decade or two, tremendous efforts have been invested into the identification of the molecular and biological processes responsible for the development of these cancers, mainly for colorectal cancer, hepatic cancer, gastric cancer, and head and neck squamous cell carcinomas (Sharma et al., 2018). In particular, identification of distinct and common features in their molecular and biological processes and also their clinical presentations can help shed light on the development and identification of diagnostic and therapeutic biomarkers.

More than 90% of the mammalian genome is transcribed into non-coding RNA (ncRNA), with a considerable number of them consisting of long ncRNAs (lncRNAs), transcripts over 200 nucleotides (nt) long (Sulayman et al., 2019). Increasing evidence has shown that lncRNAs are involved in competitive endogenous RNA (ceRNA) networks playing critical biological functions including the regulation of major cellular processes, namely, proliferation, differentiation, apoptosis, and stress response (Niu et al., 2020b; Li et al., 2021). Therefore, our study aimed to investigate the similarities and differences in the ceRNA networks of TCGA-GI cancers with more focus on lncRNAs. We included GI cancers with 11 and more than 11 matched normal tissues. These cancers were as follows: colon adenocarcinoma (COAD), rectal adenocarcinoma (READ), esophageal carcinoma (ESCA), stomach adenocarcinoma (STAD), head and neck squamous cell carcinoma (HNSC), and liver hepatocellular carcinoma (LIHC). Since HNSC is involved in the mucosa of the aero-digestive tract and also occurs in the oral cavity and salivary glands, we considered this cancer in our study as well.

Here, we report different and common shared lncRNAs and mRNAs involved in the ceRNA networks of these cancers. Moreover, our study shows the common and specific pathways in these cancers. Our finding also shows that a combination of lncRNAs and mRNA with prognostic values can promote their use for diagnostic and therapeutic aims. Furthermore, we propose a number of mRNAs that can function as new proto-oncogenes or tumor suppressors in their corresponding cancers, highlighting their diagnostic and therapeutic aspects.

Since our study reveals common shared and distinct mRNAs, lncRNAs, miRNAs, KEGG, GO-MF, GO-BP, and GO-CC among GI cancers, the identified results can help other researchers to use the data for further functional studies and differential diagnostic and prognostic strategies.

## 2 Materials and Methods

### 2.1 Data Collection and the Differential Expression Levels of lncRNAs, mRNAs, and miRNAs

The Cancer Genome Atlas (TCGA) (https://www.cancer.gov/about-nci/organization/ccg/research/structural-genomics/tcga) contains raw data of genomic, epigenomic, transcriptomic, and proteomic experiments from over 20,000 primary tumor tissues and their matched normal counterparts from 33 cancer types. In this study, we used an R/Bioconductor package, GDCRNATool developed by Ruidong Li et al. (Li et al., 2018) to download, organize and analyze lncRNAs, mRNAs, and miRNAs and clinical data of patients with GI cancers (HNSC, ESCA, STAD, LIHC, COAD, and READ) from TCGA.

Based on the following advantages, we used GDCRNATool for our analysis: One—GDCRNATools is an easy-to-use package for researchers with little coding experience. Two—It includes all data needed for performing the entire analysis smoothly in a very convenient way to download, organize, and perform comprehensive RNA expression analysis of TCGA data, with main focus on construction of the lncRNA–mRNA–miRNA-related ceRNA networks in cancer. Three—It gives not only genes involved in ceRNA networks but also all differentially expressed protein-coding genes, non-coding genes, and their GO terms and pathways. Therefore, it also has a flexibility to do other analyses that are not in the scopes of ceRNA network analysis. As an example, the differential expression data extracted from GDCRNATool can be used for considering the functional studies. Four—Moreover, to show that our differential expression data have an acceptable performance comparable to the state-of-the-art methods, we validated our expression data by looking for the expression of one gene (*UBE2C*) that was shown to be upregulated in all cancers (Dastsooz et al., 2019). Our differential expression data extracted from GDCRNATool also showed its higher expression in all these GI cancers. So, this tool can validate the differential expression data.

To validate our results, we also performed all analyses for the example that the developer of the tool did in their paper (for CHOL cancer). After confirmation of consistency between our results and theirs, we performed our analyses for GI cancers. So, we confirmed that this method of analysis is reproducible for all TCGA cancers. In our study, several analyses were carried out using GDCRNATools, which included differential gene expression analysis, univariate survival analysis, competing endogenous RNA network analysis, and functional enrichment analysis.

The appropriate data of these GI tumors and their matched adjacent non-tumor tissues were extracted. We started our analysis with the following sample numbers of RNA sequencing, miRNA, and clinical data for each cancer: COAD: 521 mRNA data, 465 miRNA data, and 459 clinical data; ESCA: 173 mRNA data, 200 miRNA data, and 185 clinical data; HNSC: 546 mRNA data, 569 miRNA data, and 528 clinical data; LIHC: 424 mRNA data, 425 miRNA data, and 377 clinical data; READ: 177 mRNA data, 165 miRNA data, and 171 clinical data; STAD: 407 mRNA data, 491 miRNA data, and 443 clinical data.

In this package, we downloaded RNA sequencing, mature miRNA, and clinical data from TCGA. Then, we parsed RNA sequencing metadata, filtered duplicated samples in this RNA metadata, and filtered non-primary tumor and non-solid tissue normal samples in them. Then, we parsed miRNAs metadata, filtered duplicated samples in these miRNAs data, and filtered non-primary tumor and non-solid tissue normal samples in the miRNAs metadata. Next, we separately merged each RNA sequencing, miRNAs, and clinical data. After that, we normalized RNA sequencing data and miRNAs data (using gdcVoomNormalization). We then used the gdcDEAnalysis function to have a whole differential expression and the gdcDEReport function for differentiation expression of each mirRNA, lncRNA, and mRNA. Using the gdcCEAnalysis function considering data of differential expression of lncRNA and protein-coding genes, and extracting data of Starbas tool, we could decipher miRNA–lncRNA and miRNA–mRNA interactions and ceRNA networks of each GI cancer.

We used this package to have the gdcParseMetadata function for the efficient organization of RNA and clinical data. With this tool, we applied the gdcFilterDuplicate function to remove duplicated samples and gdcFilterSampleType to filter out samples without primary tumors or matched normal counterparts. Raw counts data were normalized using the gdcVoomNormalization function, which applied the TMM method in edgeR and the voom method in limma. Low-expression genes with logcpm less than one were excluded from the analysis. In this R package, the gdcDEAnalysis function, which included limma, edgeR, and DESeq2, was used for the identification of differentially expressed genes (DEGs) and miRNAs between primary GI tumors and their matched normal tissues. Visualization methods were volcano, scatter, and bubble plots, and also three simple shiny apps were used in GDCRNATools. All the figures were plotted using the ggplot2 package included in this tool.

### 2.2 Construction of the ceRNA Network

Using GDCRNATool, we investigated ceRNA networks in GI cancers. This tool with its gdcCEAnalysis function considers hypergeometric test (significantly commonly shared miRNAs between lncRNA and mRNA are detected), Pearson correlation analysis (lncRNA and mRNA with positive correlations are picked up), regulation similarity analysis (commonly shared miRNAs with similar function for regulation of the lncRNA and mRNA are considered), and sensitivity Pearson partial correlation to construct ceRNA networks. Using this tool, we first extracted several miRNAs that were correlated among both the lncRNAs and mRNAs. Then, this tool identified lncRNAs and mRNAs with positive expression correlation (Pearson correlation coefficient). Finally, we gained miRNAs with similar regulative effects on lncRNAs and mRNAs. In our analysis, we applied lncTarget (to get miRNA–lncRNA interactions) and pcTarget (to consider miRNA–mRNA interactions) data along with gdcCEAnalysis function to extract miRNA–target interactions predicted from several datasets. GdcCEAnalysis function considers data of miRNA–mRNA and miRNA–lncRNA interaction databases, for example, the interaction between miRNA and mRNA and also mRNA predicted by online datasets such as Starbas. In our analysis, lncRNA–miRNA–mRNA interactions extracted as edges and nodes were visualized in Cytoscape 3.7.2.

### 2.3 mRNA–lncRNA Correlation Analysis

To find the main mRNA in each ceRNA network of GI cancers, using GDCRNA package with gdcCEAnalysis function, we also investigated lncRNAs–mRNAs correlation.

### 2.4 Survival Analysis

We performed univariate survival analysis using the gdcSurvivalAnalysis function in the GDCRNA package with the selection of Kaplan–Meier (KM) analysis. In this analysis, the patients were divided into high- and low-expression groups according to the median.

### 2.5 Pathological Tumor Stages

We looked for expression of lncRNAs and mRNAs identified in ceRNA networks of GI cancers across their pathological tumor stages using GEPIA2 web server (http://gepia2.cancer-pku.cn/#index) (Tang et al., 2019).

### 2.6 Protein–Protein Interaction Network

We used the STRING database (https://string-db.org) (Szklarczyk et al., 2019) to analyze the possible protein–protein interactions between possible novel biomarkers identified in ceRNA networks of these cancers.

### 2.7 Functional Enrichment Analysis

Using *GDCRNATool,* we investigated the role of the genes that were found to be involved in the GI cancers, in biological processes (BP), molecular functions (MF), and cellular components (CC). Using gdcEnrichAnalysis, we also carried out Gene Ontology (GO) and Kyoto Encyclopedia of Genes and Genomes (KEGG).

## 3 Results

### 3.1 Differential Expression Profile of ceRNA Network Components in GI Cancers

In the current study, we found different numbers of up- and downregulated mRNAs, lncRNAs, and miRNAs in HNSC, ESCA, STAD, COAD, READ, and LIHC given in Table 1. Regarding COAD, these deferentially expression numbers and the lncRNAs were also previously listed (Poursheikhani et al., 2020). Regarding STAD and LIHC, some deferentially expression numbers have been reported by Zhang et al. (2020a) and Zheng and Yu (2021), respectively.

**TABLE 1:** Number of differentially expressed mRNAs, lncRNAs, and miRNAs in GI cancers.

Tumor type	COAD		READ		STAD		HNSC		LIHC		ESCA	
RNA type	Up	Down	Up	Down	Up	Down	Up	Down	Up	Down	Up	Down
mRNA	1,094	1,901	1,169	1,790	935	1,192	940	1,103	715	1,448	610	722
lncRNA	128	77	181	53	119	51	76	32	68	80	49	49
miRNA	170	160	165	114	67	59	88	81	59	71	46	33

### 3.2 ceRNA Networks in GI Cancers

Our study revealed the following numbers of differentially expressed mRNAs (DEmRNAs), lncRNA (DElncRNA), and miRNAs (DemiRNAs) maintained in the ceRNA networks of each cancer (Supplementary Table S1): HNSC (6 lncRNAs, 30 miRNAs, and 64 mRNAs), ESCA (4 lncRNAs, 17 miRNAs, and 15 mRNAs), COAD [9 lncRNAs, 37 miRNAs, and 71 mRNAs, the ceRNA network for COAD cancer was previously shown in a different way (Poursheikhani et al., 2020)], STAD (4 lncRNAs, 18miRNAs, and 29 mRNAs), READ (9 lncRNAs, 21miRNAs, and 45 mRNAs), and LIHC [10 lncRNAs, 30 miRNAs, and 64 mRNAs, its ceRNA network was shown in a different figure by Zheng and Yu (2021)] (Supplementary Table S1).

**FIGURE 1:**
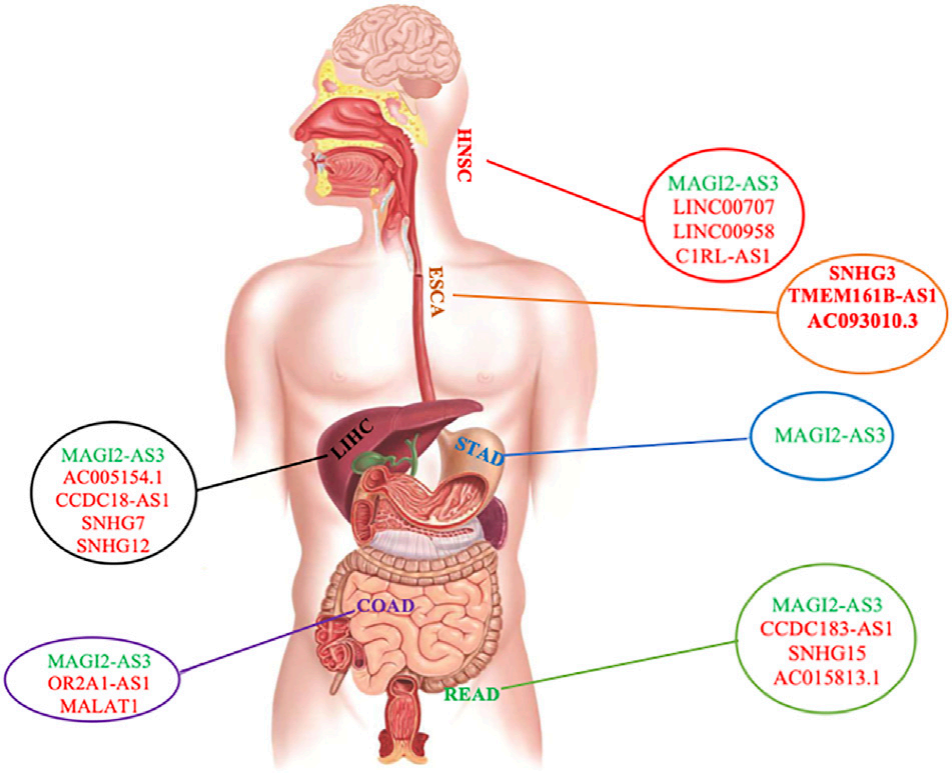
Distribution of common and specific lncRNAs among different GI cancers. lncRNA in green color is the common one among GI cancers. lncRNAs in red are specific in each GI cancer. The gastrointestinal tract shown in this figure was reproduced from an image published under creative commons attribution license (Iriondo-Dehond et al., 2020).

Our findings showed that LIHC, COAD, and READ had more ceRNA networks. It is worth noting that our study revealed *MAGI2-AS3* lncRNA to be shared among almost all ceRNA networks of GI cancers, except for ESCA, indicating the involvement of the same pathways in GI cancers (Supplementary Table S2, Supplementary Figure S1, Figure 1). Furthermore, *PVT1* was common between LIHC, ESCA, and STAD; *GAS5*, *SNHG1*, and *SNHG20* were common between LIHC, COAD, and READ; *KCNQ1OT1* was common between STAD, HNSC, COAD, and READ; *H19* was found to be common between LIHC, HNSC, and COAD; and finally, *MIR17HG* was common between STAD, COAD, and READ. We also investigated commonly shared lncRNAs between COAD and READ (CRCs) and found the presence of *MAGI2-AS3*, *GAS5*, *SNHG1*, *KCNQ1OT1*, *SNHG20*, and *MIR17HG* in both cancers. We were also able to distinguish COAD and READ using differential expression of *H19*, *OR2A1-AS1*, and *MALAT1* (specific for COAD) and *SNHG15*, *AC015813.1*, and *CCDC183-AS1* (specific for READ) (Supplementary Table S2 and Supplementary Figure S1).

For each cancer ceRNA network, we found that the expression level of a number of lncRNAs was specific to each cancer: *SNHG12*, *AC005154.1*, *CCDC18-AS1*, and *SNHG7* were specific for LIHC; *SNHG3*, *TMEM161B-AS1*, and *AC093010.3* were specific for ESCA; *SNHG15*, *AC015813.1*, and *CCDC183-AS1* were specific for READ; *C1RL-AS1*, *LINC00707*, and *LINC00958* were specific for HNSC; *OR2A1-AS1* and *MALAT1* were specific for COAD (Figure 1 and Supplementary Table S2). There was no specific lncRNA for ceRNA network of STAD. However, in comparison with other GI cancers, it was possible to distinguish it from other cancers (Figure 1, Supplementary Table S2, and Supplementary Figure S1).

Our study revealed that ceRNA networks with involvements of the following lncRNAs consist of a number of important mRNAs, indicating the presence of important ceRNA networks in their corresponding cancers: *MAGI2-AS3, SNHG15, KCNQ1OT1, SNHG1,* and *MIR17HG* in READ; *MAGI2-AS3, KCNQ1OT1, H19, MIR17HG, SNHG1,* and *MALAT1* in COAD; *MAGI2-AS3, KCNQ1OT1,* and *MIR17HG* in STAD; *MAGI2-AS3*, *PVT1*, *AC005154.1*, *H19*, *CCDC18*, and *SNHG1* in LIHC; *MAGI2-AS3, KCNQ1OT1, H19, CIRL-AS1, LINC00707,* and *LINC00958* in HNSC; and finally *TMEM161B-AS1* and *AC093010.3* in ESCA (Supplementary Figure S1).

We mentioned that *MAGI2-AS3* is shared in approximately all GI cancers. Then, we looked for proteins involved in its network and its significant interaction with them. We found several important ones shown in Supplementary Table S3. In all *MAGI2-AS3* networks, it has interacted with the following miRNAs: *has-miR-374a-5p* and *has-miR-374b-5p.* Its main mRNA partners that have positive correlations are *MEIS1, PPP1R3C, ADAMTSL3, RIPOR2,* and *MYLK.* It seems that the function of *MAGI2-AS3* is mainly dependent on its interaction with these five positive correlated genes in COAD, READ, and STAD. However, in LIHC, it is dependent on *ADAMTSL3, RIPOR2*, and *MYLK*, and in HNSC, it is dependent on the interaction with *MEIS1* and *PPP1R3C* (Supplementary Table S3 and Supplementary Figure S1)*.*

To find shared mRNAs among ceRNA networks of GI cancers, we compared the networks and identified four mRNAs, which were common in most of them as follows: *MEIS1* and *PPP1R3C* among CC-1 (COAD, READ, STAD, and HNSC), and *ADAMTSL3* and *MYLK* among CC-2 (COAD, READ, STAD, and LIHC) (Table 2 and Supplementary Figure S1). Moreover, we found several important shared mRNAs between ceRNA networks of COAD and READ (CRC, cancer cluster 3:CC-3) (Table 2), which included *APC*, *EDIL3*, *FGFR2*, *HOMER1*, *MIER3*, *PI15*, *RBM28*, *RRS1*, *SCML1*, *SGPP1*, *TOMM34*, *TRAF5*, *WNT5A*, and *ZNF655*. For other cancer clusters, other important shared mRNAs as shown in Table 2 were identified. These data indicated the common pathways for ceRNA networks of each cancer cluster. However, our data also showed specific protein-coding genes for each cancer presented in Supplementary Table S2, demonstrating specific ceRNA pathways for each cancer.

**TABLE 2:** Commonly shared mRNAs between ceRNA networks of GI cancers.

CC-1	*MEIS1* and *PPP1R3C*
CC-2	*ADAMTSL3* and *MYLK*
CC-3	*APC, EDIL3, FGFR2, HOMER1, MIER3, PI15, RBM28, RRS1, SCML1, SGPP1, TOMM34, TRAF5, WNT5A,* and *ZNF655*
CC-4	*ATP8B2, L1CAM, LPAR1, NTN1, PCDH7, TTLL7, UST,* and *VAMP2*
CC-5	*ANKRD13B* and *RIPOR2*
CC-6	*CDC7, SOX12,* and *SRPX*
CC-7	*BUB1* and *LMNB2*
CC-8	*COL5A2, DI O 2, KDELC1, SPARC,* and *STX1A*
CC-9	*NFIX* and *PDZD2*
CC-10	*SPRY2*
CC-11	*RPS6KA5* (ESCA and READ) and *DAAM2* (ESCA and COAD)

CC-1: mRNAs shared between ceRNA networks of COAD, READ, HNSC, and STAD; CC-2: mRNA shared between ceRNA networks of COAD, READ, LIHC, and STAD; CC-3: mRNAs shared between ceRNA networks of COAD, and READ (CRCs); CC-4: mRNAs shared between ceRNA networks of STAD, and CRCs; CC-5: mRNA shared between ceRNA networks of LIHC, and CRCs, CC-6: mRNAs shared between ceRNA networks of LIHC, and COAD; CC-7: mRNAs shared between ceRNA networks of LIHC, and READ; CC-8: mRNAs shared between ceRNA networks of HNSC, and COAD; CC-9: mRNAs shared between ceRNA networks of HNSC, and STAD; CC-10: mRNAs shared between ceRNA networks of HNSC, and LIHC; C11: mRNAs shared between ceRNA networks of ESCA, and other GI, cancers.

### 3.3 OS Analysis

**FIGURE 2:**
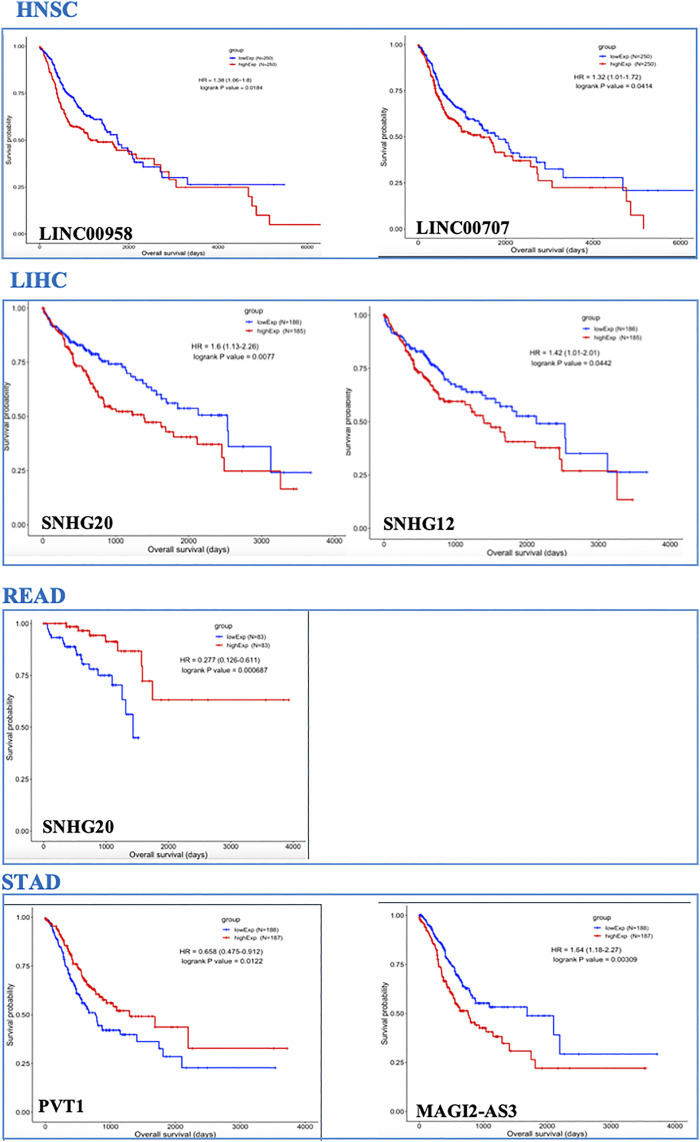
OS analysis for lncRNAs in TCGA-GI cancers. Only lncRNAs with prognostic values are shown.

In the GI-ceRNA networks, we identified that the overexpression of *LINC00958* and *LINC00707* in HNSC and overexpression of *SNHG20* and *SNHG12* in LIHC were correlated with reduced OS and worse prognosis. However, overexpression of *SNHG20* in READ and *PVT1* in STAD and low expression of *MAGI2-AS3* in STAD were correlated with good prognosis (Figure 2). Moreover, OS analysis for mRNAs involved in ceRNA networks of these cancers showed prognostic values of several mRNAs given in Table 3 and Supplementary Figure S2. Among them, we identified some new prognostic biomarkers (reduced OS with worse prognosis) that have not been reported or supported by functional studies in their corresponding tumors as follows: upregulation of *KDELC1* (*POGLUT2*) in HNSC, downregulation of *RIPOR2* and *SMOC1*, and upregulation of *TMEM164*, *B3GNT5*, *ZNF607*, and *ANKRD13B* in LIHC. These genes showed reduced OS with worse prognosis. The downregulated genes with reduced OS and worse prognosis may function as tumor suppressors, but those overexpressed mRNAs with reduced OS and worse prognosis can act as proto-oncogenes.

**TABLE 3:** mRNAs as prognostic biomarkers identified in ceRNA networks of TCGA-GI cancers.

TCGA cancer type	Gene	Worse prognosis	Good prognosis
COAD	*HOMER1*		Over-expression
	*NRG1*	Low expression	
HNSC	*KDELC1, GNA12, ITGA5*, and *CDCA4*	Over-expression	
	*MEIS1*	Low expression	
LIHC	*RUNX3, CD69, RIPOR2 (FAM65B), GADD45A, SYBU, ADAMTSL3, AUTS2, SMOC1*	Low expression	
	*SCML2, LIMK1, FANCE, PLXNA1, E2F8, EFNA3, SLC7A11, ENAH, TMEM164, H2AFZ, BUB1, CBX2, EXO1, LPL, B3GNT5, LMNB2, SOX12, JPT1, MAFG, ZNF607, STK39,* and *ANKRD13B*	Over-expression	
	*CDC7* and *PFKFB3*		Low expression
	*DNM3* and *COL15A1*		Over-expression
READ	*ATP8B2, NTN1, NOVA1,* and *PCDH7*		Low expression
STAD	*MAP7*		Over-expression

It worth noting that for LIHC, combinations of prognostic values of *SNHG20* lncRNA and *BUB1* mRNA, and also *SNHG12* lncRNA and *TMEM164* mRNA (associated with reduced OS with worse prognosis), could strengthen the predictive value of these markers for this cancer. Each of the lncRNA–mRNA combinations was in the same ceRNA network.

### 3.4 Pathological GI Tumor Stages

**FIGURE 3:**
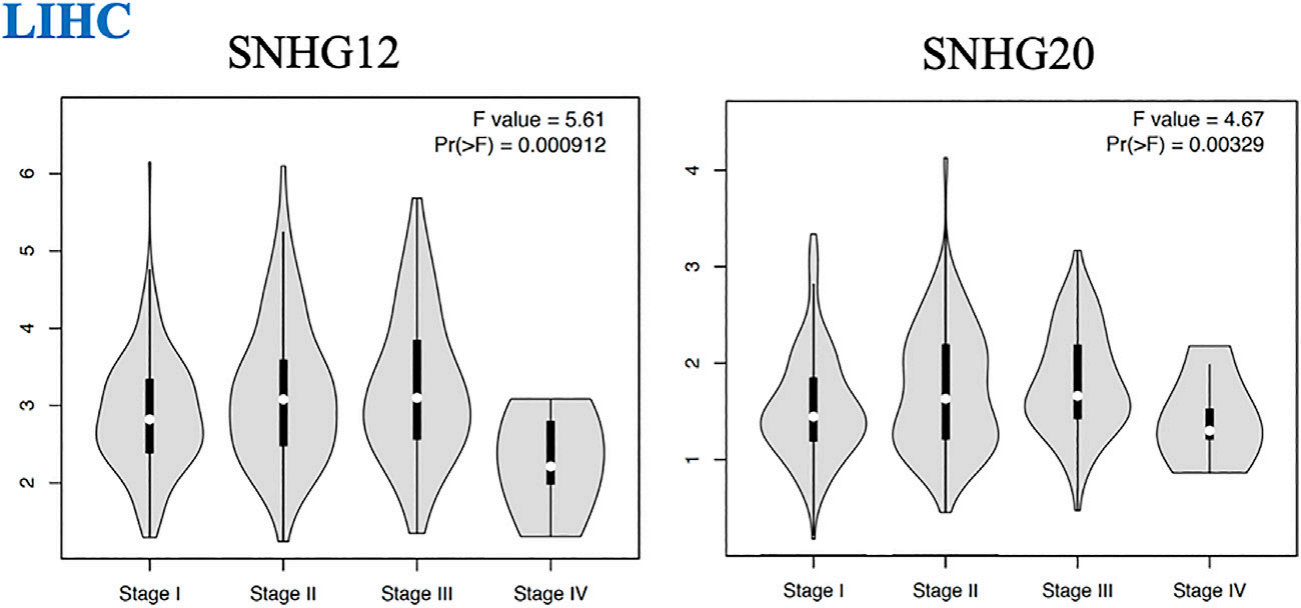
Pathological tumor stages of LIHC and lncRNAs with significant altered expression across different stages of this malignancy.

We found that the expression of some lncRNAs with worse prognosis in ceRNA networks of GI cancers had significant alteration across cancer stages, indicating their roles in cancer progression and invasion. These lncRNAs were *SNHG12* and *SNHG20* in LIHC (Figure 3).

In addition to the lncRNAs, we found that several mRNA with worse prognosis in their corresponding networks showed significant altered expression across tumor stages, indicating their roles in cancer progression and invasion. These genes were as follows: *LIMK1* (*p* = 0.000934), *PLXNA1* (*p* = 0.00176)*, E2F8* (*p* = 0.0155), *EFNA3* (*p* = 3.74e−07), *SLC7A11* (*p* = 0.0497), *TMEM164* (*p* = 0.00469), *H2AFZ* (*p* = 5.27e−06), *BUB1* (*p* = 5.82e−06), *CBX2* (*p* = 0.00033), *EXO1* (*p* = 4.88e−06), *FANCE* (*p* = 0.000301), *LMNB2* (*p* = 0.000311), *SOX12* (*p* = 0.00467), *JPT1* (*p* = 4.08e−07), *MAFG* (*p* = 0.00305), *STK39* (*p* = 0.0428), *ANKRD13B* (*p* = 0.000412) (Supplementary Figure S3).

### 3.5 Providing Evidence That Highlights the Need for Functional Study on the Role of *KDELC1* in Cancers

**FIGURE 4:**
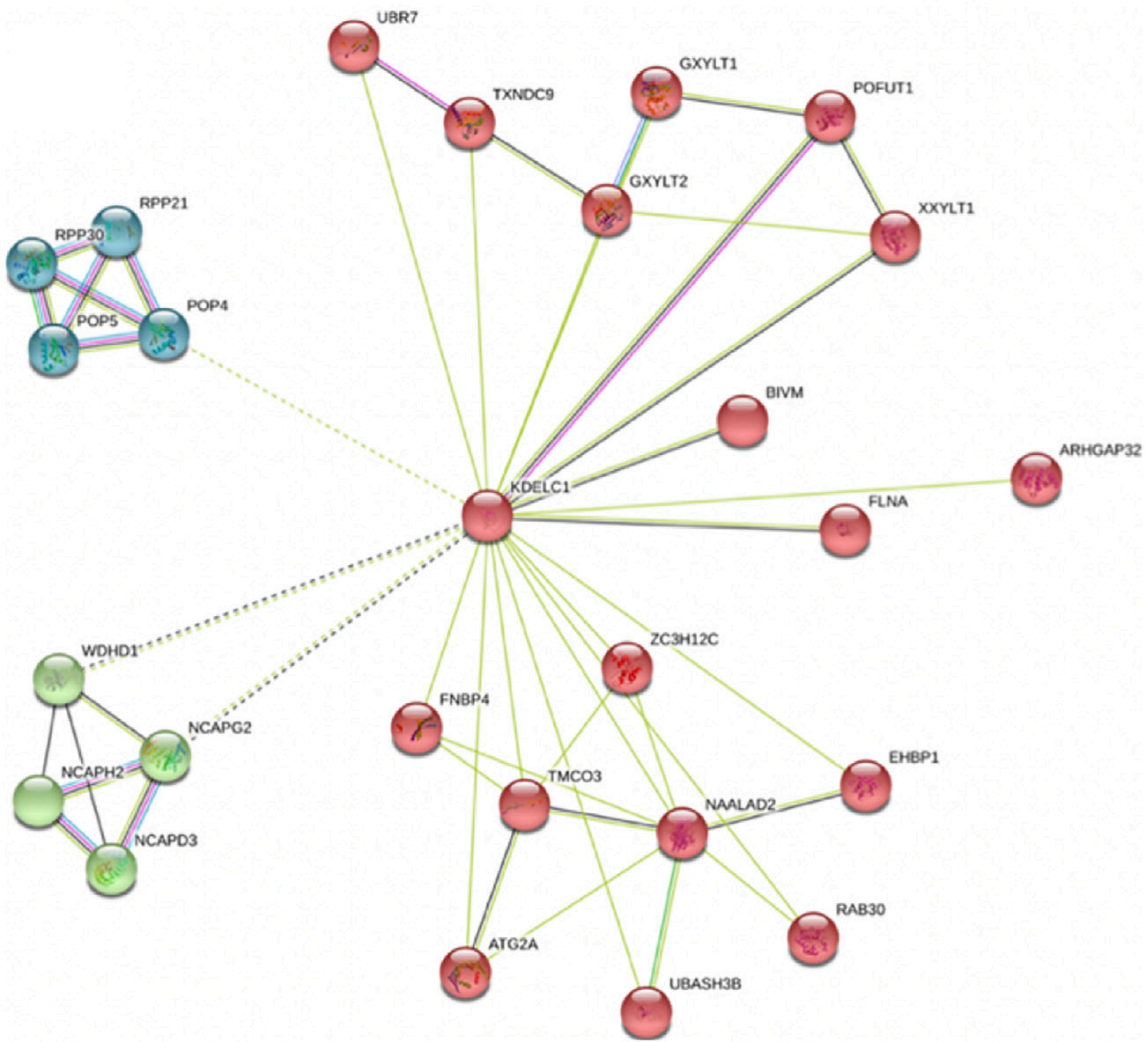
KDELC1 protein network extracted from STRING webserver.

The role of *RIPOR2, SMOC1, B3GNT5, ANKRD13B*, and *TMEM164* has not been clearly demonstrated in LIHC. Moreover, the role of *ZNF607* and mainly *KDELC1* has not been clearly delineated in any cancer [*in-silico* analysis by Zhou et al. (2021)]. However, our findings in this study highlight the need for functional study of *KDELC1* in cancers as follows: One—Our data showed its over-expression with worse prognosis (reduced OS) in HNSC. Two—It showed overexpression in several tumors such COAD, kidney renal clear cell carcinoma (KIRC), and kidney renal papillary cell carcinoma (KIRP) and lower expression in Kidney Chromophobe (KICH) (Table 4). Three—It is mainly localized in the nucleus (confidence score: 5) and endoplasmic reticulum (confidence score: 5), followed by cytosol (confidence score: 4) (https://www.genecards.org and https://www.proteinatlas.org). Four—It specifically targets extracellular EGF repeats of important proteins such as NOTCH1 and NOTCH3 (Takeuchi et al., 2018), indicating its possible roles in the Notch signaling pathway. Five—Using STRING database, we found that it had interactions with several proteins involved in different cancers such as *TXNDC9* (Feng et al., 2020b), *GXYLT2* (Cui et al., 2019), *POFUT1* (Chabanais et al., 2018 *)*, *XXYLT1(* Zeng et al., 2021 *)*, *FLNA* (Guo et al., 2018), and *ZC3H12C* (Suk et al., 2018) (Figure 4). These evidences strengthen the possible important role of *KDELC1* in various malignancies*.*

**TABLE 4:** *KDELC1* over-expression in TCGA cancers.

Cancer type	logFC	Average expression	*p*-value	Fdr
HNSC	1.4	2.62	7.05E-19	1.25E-17
COAD	1.1	2.38	1.79E-16	8.36E-16
KIRC	1.1	3.72	3.40E-24	1.61E-23
KIRP	1.95	4.35	5.58E-14	5.14E-13
KICH	-1.6	1.5	1.27E-16	2.18E-15

### 3.6 Functional Enrichment Analysis

In our study, firstly, we extracted the 10 top GO terms for BP, CC, and MF in the TCGA-GI cancers. Then, we compared these GO terms between GI cancers (Supplementary Figure S4 and Supplementary Table S4). Our study revealed some common and specific GO terms for these cancers given in Supplementary Tables S5,6, respectively. These terms were previously shown in COAD cancer (Poursheikhani et al., 2020); however, in our study, we looked for common and specific terms in GI cancers. For MF, our study revealed extracellular matrix structural constituent (GO:0,005,201) as the top shared term among all GI cancers (Supplementary Table S5). In relation to the CC, we identified collagen-containing extracellular matrix (GO:0062023), which was shared between all GI cancers (Supplementary Table S5). For BP, the most significantly shared GO terms in these cancers were extracellular matrix organization (GO:0030198), which was shared among all studied malignancies except LIHC. In addition, regulation of chromosome segregation (GO:0051983) and mitotic nuclear division (GO:0140014) were shared among all investigated cancers excepting READ (Supplementary Table S5). Regarding KEGG, we found Cell cycle (hsa04110) as the top shared one among all GI cancers (Supplementary Table S5). However, we found that some BP, CC, and MF terms were exclusively specific for each cancer (Supplementary Table S6).

## 4 Discussion

Our study revealed differential expression of mRNAs, lncRNAs, and miRNAs in ceRNA networks of GI cancers. We highlighted the important function of several lncRNAs and mRNAs specific for each cancer or commonly shared between GI cancers, which might be used for diagnostic and therapeutic purposes.

Our study showed that *MAGI2-AS3* lncRNAs were common between most of these cancers, representing possible involvement of the same pathways in different GI cancers. In ceRNA networks of each cancer, we also found that deferential expression of some lncRNAs was specific to each cancer.

We found several lncRNAs as prognostic biomarkers including *LINC00958* and *LINC00707* in HNSC; *SNHG20* and *SNHG12* in LIHC (correlated with reduced OS with worse prognosis); *SNHG20* in READ; and *PVT1* and *MAGI2-AS3* in STAD (correlated with good prognosis). We found that over-expression of *SNHG12* and *SNHG20* in LIHC (Figure 3) had significant alteration across cancer stages, representing their roles in cancer progression and invasion. These lncRNAs identified in our study were previously reported in their corresponding cancers (Yang et al., 2015; Zhang et al., 2016; Li et al., 2019; Tamang et al., 2019; Feng et al., 2020a; Niu et al., 2020a; Martinez-Barriocanal et al., 2020; Shen et al., 2020; Zeng et al., 2020; Zhang et al., 2020b; Zheng et al., 2020) but a comprehensive investigation of distinct or shared lncRNAs in these cancers has not yet been conducted.

Notably, we found a number of mRNAs with prognostic values (reduced OS with worse prognosis), which were not previously reported or supported by functional studies in their corresponding human tumors as follows: over-expression of *KDELC1* in HNSC; over-expression of *TMEM164*, *B3GNT5*, *ZNF607*, and *ANKRD13B*; and under-expression of *RIPOR2* and *SMOC1* in LIHC. The role of *ZNF607* and mainly *KDELC1* has not been clearly elucidated in cancers. Among these mRNAs, *TMEM164, ZNF607,* and *ANKRD13B* showed significant altered expression across tumor stages, indicating their role in cancer progression and invasion. Therefore, functional studies focused on these prognostic biomarkers in their corresponding cancers are suggested. Moreover, among GI cancers, four mRNAs were more common in most of them, which included *MEIS1* and *PPP1R3C* among COAD, READ, STAD, and HNSC; and *ADAMTSL3* and *MYLK* among COAD, READ, STAD, and LIHC. Furthermore, we found several important shared mRNAs between ceRNA networks of COAD and READ (Table 2, CRC, cancer cluster 3:CC-3).

According to the findings of the current study, we strongly proposed a novel proto-oncogene, *KDELC1* (*POGLUT2*), in cancers since it is mainly localized in nucleus and endoplasmic reticulum, it specifically targets extracellular EGF repeats of important proteins involved in Notch signaling pathways, it has interactions with several main proteins involved in cancers, and it is upregulated in several TCGA cancers. Moreover, our study revealed several mRNAs associated with worse prognosis in cancer and showed their significant altered expression across tumor stages.

Our study found that in ceRNA networks of LIHC, combination of prognostic values of *SNHG20* lncRNA and *BUB1* mRNA, and also *SNHG12* lncRNA and *TMEM164* mRNA (associated with reduced OS with worse prognosis), could be used as collective prognostic values for this cancer.

In this study, we found extracellular matrix structural constituent as the top shared MF term, collagen-containing extracellular matrix as the top shared CC, and Cell cycle (hsa04110) as the top shared KEGG among all these GI cancers. However, extracellular matrix organization, except for LIHC, and regulation of chromosome segregation and mitotic nuclear division, both except for READ, were found to be the most significantly shared BP terms among the GI cancers.

In conclusion, we found several common and distinct prognostic mRNAs and lncRNAs in GI cancers. The most important finding was the involvement of *MAGI2-AS3* lncRNA in almost all GI cancers. Another interesting result was the identification of the most common shared mRNAs in these cancers*.* Moreover, our study revealed a number of specific lncRNAs and mRNAs in GI cancers. Furthermore, our study reported several genes, most notably *KDELC1*, that can have proto-oncogenic roles in cancers. In addition to the abovementioned findings, we reported the top shared KEGG, GO-MF, GO-BP, and GO-CC among GI cancers. Overall, our study showed that bioinformatic studies regarding mRNA-miRNA–lncRNA networks of TCGA data can help identify prognostic biomarkers. The identified common shared and distinct mRNAs, lncRNAs, miRNAs, KEGG, and GO-terms among GI cancers can be used by other researchers for further functional experiments and also for diagnostic and prognostic approaches.

## Data Availability

Publicly available datasets were analyzed in this study include TCGA, GEPIA2, STRING, Genecards, and Human Protein Atlas. The raw data can be found here: https://tcga‐data.nci.nih.gov/tcga/, http://gepia2.cancer-pku.cn/#index, https://string-db.org, https://www.genecards.org, and https://www.proteinatlas.org.

## Supplementary Material

Supplementary Figure S1 ceRNA networks of LIHC, HNSC, READ, and ESCA.

Supplementary Figure S2 OS analysis of protein-coding genes in patients with TCGA-GI cancers (HNSC, LIHC, READ, STAD, COAD).

Supplementary Figure S3 mRNAs with significant altered expression across tumor stages in ceRNA networks of GI cancers.

Supplementary Figure S4 GSEA of GI cancers (COAD, ESCA, HNSC, LIHC, READ, STAD).

Supplementary Table S1 Differentially expressed mRNAs, lncRNA, and miRNAs in the ceRNA networks of GI cancers.

Supplementary Table S2 Common and distinct lncRNAs and specific mRNAs in ceRNA networks of GI cancers.

Supplementary Table S3 Protein-coding genes involved in MAGI2-AS3 ceRNA network of GI cancers.

Supplementary Table S4 The GO terms (BP, CC, and MF) and KEGG pathways involved in the TCGA-GI cancers.

Supplementary Table S5 Common GO terms and KEGG pathways of the GI cancers.

Supplementary Table S6 Specific and distinct GO terms and KEGG pathways of the GI cancers.

## References

[B1] Arnold M. Abnet C. C. Neale R. E. Vignat J. Giovannucci E. L. Mcglynn K. A. (2020). Global Burden of 5 Major Types of Gastrointestinal Cancer. Gastroenterology 159, 335–349. e315. 10.1053/j.gastro.2020.02.068 32247694 PMC8630546

[B2] Chabanais J. Labrousse F. Chaunavel A. Germot A. Maftah A. (2018). POFUT1 as a Promising Novel Biomarker of Colorectal Cancer. Cancers (Basel) 10, 1. 10.3390/cancers10110411 PMC6266312 30380753

[B3] Cui Q. Xing J. Gu Y. Nan X. Ma W. Chen Y. (2019). GXYLT2 Accelerates Cell Growth and Migration by Regulating the Notch Pathway in Human Cancer Cells. Exp. Cel Res. 376, 1–10. 10.1016/j.yexcr.2019.01.023 30716301

[B4] Dastsooz H. Cereda M. Donna D. Oliviero S. (2019). A Comprehensive Bioinformatics Analysis of UBE2C in Cancers. Int. J. Mol. Sci. 20, 1. 10.3390/ijms20092228 PMC6539744 31067633

[B5] Feng L. Li H. Li F. Bei S. Zhang X. (2020a). LncRNA KCNQ1OT1 Regulates microRNA-9-Lmx1a Expression and Inhibits Gastric Cancer Cell Progression. Aging 12, 707–717. 10.18632/aging.102651 31915311 PMC6977675

[B6] Feng T. Zhao R. Sun F. Lu Q. Wang X. Hu J. (2020b). TXNDC9 Regulates Oxidative Stress-Induced Androgen Receptor Signaling to Promote Prostate Cancer Progression. Oncogene 39, 356–367. 10.1038/s41388-019-0991-3 31477836

[B7] Guo Y. Li M. Bai G. Li X. Sun Z. Yang J. (2018). Filamin A Inhibits Tumor Progression through Regulating BRCA1 Expression in Human Breast Cancer. Oncol. Lett. 16, 6261–6266. 10.3892/ol.2018.9473 30405761 PMC6202495

[B8] Iriondo-Dehond A. Uranga J. A. Del Castillo M. D. Abalo R. (2020). Effects of Coffee and its Components on the Gastrointestinal Tract and the Brain-Gut Axis. Nutrients 13, 1. 10.3390/nu13010088 PMC7824117 33383958

[B9] Li R. Qu H. Wang S. Wei J. Zhang L. Ma R. (2018). GDCRNATools: an R/Bioconductor Package for Integrative Analysis of lncRNA, miRNA and mRNA Data in GDC. Bioinformatics 34, 2515–2517. 10.1093/bioinformatics/bty124 29509844

[B10] Li S. J. Wang L. Sun Z. X. Sun S. J. Gao J. Ma R. L. (2019). LncRNA SNHG1 Promotes Liver Cancer Development through Inhibiting P53 Expression via Binding to DNMT1. Eur. Rev. Med. Pharmacol. Sci. 23, 2768–2776. 10.26355/eurrev_201904_17550 31002127

[B11] Li Z. Wang F. Zhu Y. Guo T. Lin M. (2021). Long Noncoding RNAs Regulate the Radioresistance of Breast Cancer. Anal. Cel Pathol (Amst) 2021, 9005073. 10.1155/2021/9005073 PMC8478560 34595090

[B12] Martínez-Barriocanal Á. Arango D. Dopeso H. (2020). PVT1 Long Non-coding RNA in Gastrointestinal Cancer. Front. Oncol. 10, 38. 10.3389/fonc.2020.00038 32083000 PMC7005105

[B13] Niu J. Song X. Zhang X. (2020a). Regulation of lncRNA PVT1 on miR-125 in Metastasis of Gastric Cancer Cells. Oncol. Lett. 19, 1261–1266. 10.3892/ol.2019.11195 31966056 PMC6956415

[B14] Niu Z.-S. Wang W.-H. Dong X.-N. Tian L.-M. -L. (2020b). Role of Long Noncoding RNA-Mediated Competing Endogenous RNA Regulatory Network in Hepatocellular Carcinoma. Wjg 26, 4240–4260. 10.3748/wjg.v26.i29.4240 32848331 PMC7422540

[B15] Poursheikhani A. Abbaszadegan M. R. Nokhandani N. Kerachian M. A. (2020). Integration Analysis of Long Non-coding RNA (lncRNA) Role in Tumorigenesis of colon Adenocarcinoma. BMC Med. Genomics 13, 108. 10.1186/s12920-020-00757-2 32727450 PMC7392656

[B16] Sharma K. L. Bhatia V. Agarwal P. Kumar A. (2018). Gastrointestinal Cancers: Molecular Genetics and Biomarkers. Can. J. Gastroenterol. Hepatol. 2018, 4513860. 10.1155/2018/4513860 30941325 PMC6420989

[B17] Shen A. Ma J. Hu X. Cui X. (2020). High Expression of lncRNA-SNHG7 Is Associated with Poor Prognosis in Hepatocellular Carcinoma. Oncol. Lett. 19, 3959–3963. 10.3892/ol.2020.11490 32382340 PMC7202315

[B18] Suk F. M. Chang C. C. Lin R. J. Lin S. Y. Chen Y. T. Liang Y. C. (2018). MCPIP3 as a Potential Metastasis Suppressor Gene in Human Colorectal Cancer. Int. J. Mol. Sci. 19, 1. 10.3390/ijms19051350 PMC5983627 29751537

[B19] Sulayman A. Tian K. Huang X. Tian Y. Xu X. Fu X. (2019). Genome-wide Identification and Characterization of Long Non-coding RNAs Expressed during Sheep Fetal and Postnatal Hair Follicle Development. Sci. Rep. 9, 8501. 10.1038/s41598-019-44600-w 31186438 PMC6559957

[B20] Szklarczyk D. Gable A. L. Lyon D. Junge A. Wyder S. Huerta-Cepas J. (2019). STRING V11: Protein-Protein Association Networks with Increased Coverage, Supporting Functional Discovery in Genome-wide Experimental Datasets. Nucleic Acids Res. 47, D607–D613. 10.1093/nar/gky1131 30476243 PMC6323986

[B21] Takeuchi H. Schneider M. Williamson D. B. Ito A. Takeuchi M. Handford P. A. (2018). Two Novel Protein O-Glucosyltransferases that Modify Sites Distinct from POGLUT1 and Affect Notch Trafficking and Signaling. Proc. Natl. Acad. Sci. USA 115, E8395–E8402. 10.1073/pnas.1804005115 30127001 PMC6130362

[B22] Tamang S. Acharya V. Roy D. Sharma R. Aryaa A. Sharma U. (2019). SNHG12: An LncRNA as a Potential Therapeutic Target and Biomarker for Human Cancer. Front. Oncol. 9, 901. 10.3389/fonc.2019.00901 31620362 PMC6759952

[B23] Tang Z. Kang B. Li C. Chen T. Zhang Z. (2019). GEPIA2: an Enhanced Web Server for Large-Scale Expression Profiling and Interactive Analysis. Nucleic Acids Res. 47, W556–W560. 10.1093/nar/gkz430 31114875 PMC6602440

[B24] Yang M.-H. Hu Z.-Y. Xu C. Xie L.-Y. Wang X.-Y. Chen S.-Y. (2015). MALAT1 Promotes Colorectal Cancer Cell Proliferation/migration/invasion via PRKA Kinase Anchor Protein 9. Biochim. Biophys. Acta (Bba) - Mol. Basis Dis. 1852, 166–174. 10.1016/j.bbadis.2014.11.013 PMC4268411 25446987

[B25] Zeng H. Wang Y. Wang Y. Zhang Y. (2021). XXYLT1 Methylation Contributes to the Occurrence of Lung Adenocarcinoma. Medicine (Baltimore) 100, e24150. 10.1097/md.0000000000024150 33429795 PMC7793369

[B26] Zeng J. Liu Z. Zhang C. Hong T. Zeng F. Guan J. (2020). Prognostic Value of Long Non-coding RNA SNHG20 in Cancer. Medicine (Baltimore) 99, e19204. 10.1097/md.0000000000019204 32118721 PMC7478608

[B27] Zhang D. Cao C. Liu L. Wu D. (2016). Up-regulation of LncRNA SNHG20 Predicts Poor Prognosis in Hepatocellular Carcinoma. J. Cancer 7, 608–617. 10.7150/jca.13822 27053960 PMC4820738

[B30] Zhang X. Zheng P. Li Z. Gao S. Liu G. (2020a). The Somatic Mutation Landscape and RNA Prognostic Markers in Stomach Adenocarcinoma. Onco Targets Ther. 13, 7735–7746. 10.2147/OTT.S263733 32801780 PMC7414981

[B28] Zhang Y. Huang W. Yuan Y. Li J. Wu J. Yu J. (2020b). Long Non-coding RNA H19 Promotes Colorectal Cancer Metastasis via Binding to hnRNPA2B1. J. Exp. Clin. Cancer Res. 39, 141. 10.1186/s13046-020-01619-6 32698890 PMC7412843

[B29] Zheng X. Wang X. Zheng L. Zhao H. Li W. Wang B. (2020). Construction and Analysis of the Tumor-specific mRNA-miRNA-lncRNA Network in Gastric Cancer. Front. Pharmacol. 11, 1112. 10.3389/fphar.2020.01112 32848739 PMC7396639

[B31] Zheng C. Yu S. (2021). Expression and Gene Regulatory Network of SNHG1 in Hepatocellular Carcinoma. BMC Med Genomics 14 (1), 28. 10.1186/s12920-021-00878-2 33499863 PMC7836560

[B32] Zhou H. He Y. Li L. Wu C. Hu G. (2021). Identification Novel Prognostic Signatures for Head and Neck Squamous Cell Carcinoma Based on ceRNA Network Construction and Immune Infiltration Analysis. Int. J. Med. Sci. 18 (5), 1297–1311. 10.7150/ijms.53531 33526991 PMC7847625

