# Supplementary material for: LncRNA–miRNA–mRNA Networks of Gastrointestinal Cancers Representing Common and Specific LncRNAs and mRNAs
Source: Front Genet. 2022 Jan 24;12:791919. doi: 10.3389/fgene.2021.791919 (PMC8819090; doi:10.3389/fgene.2021.791919)
Supplement: Supplementary file 3 [file Image4.pdf]

Supplementary Figure 4. GSEA of GI cancers. A: COAD, B: ESCA, C: HNSC, D: LIHC, E: READ, F: STAD.

(A) COAD

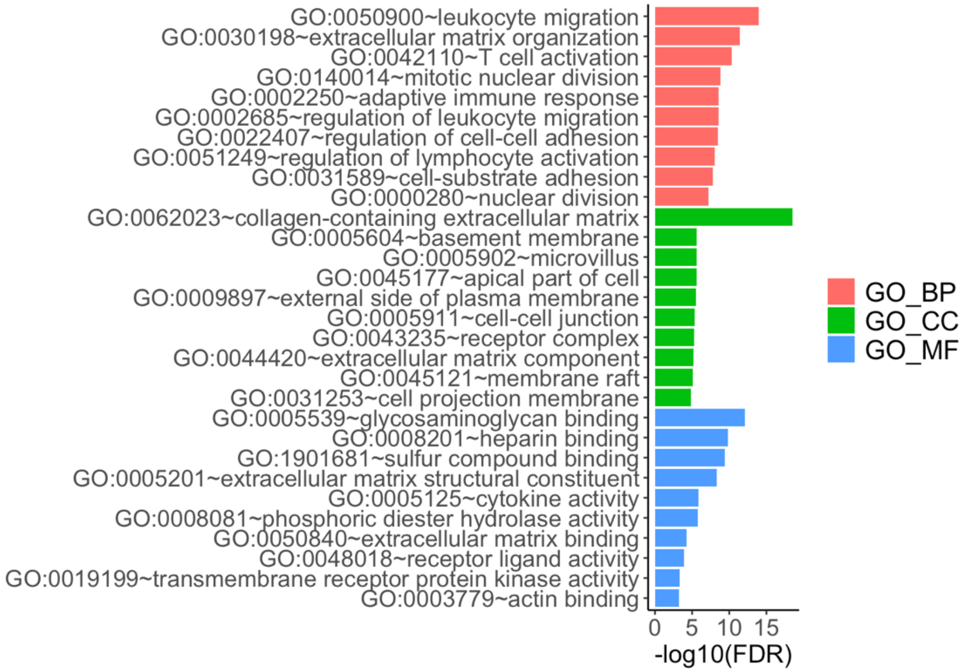

(B) ESCA

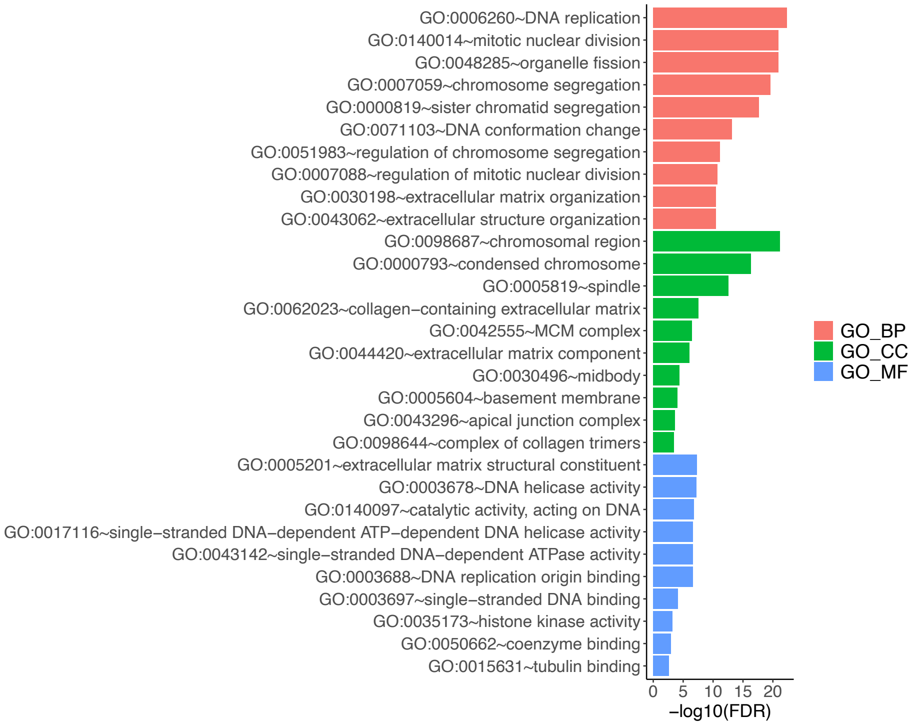

(C) HNSC

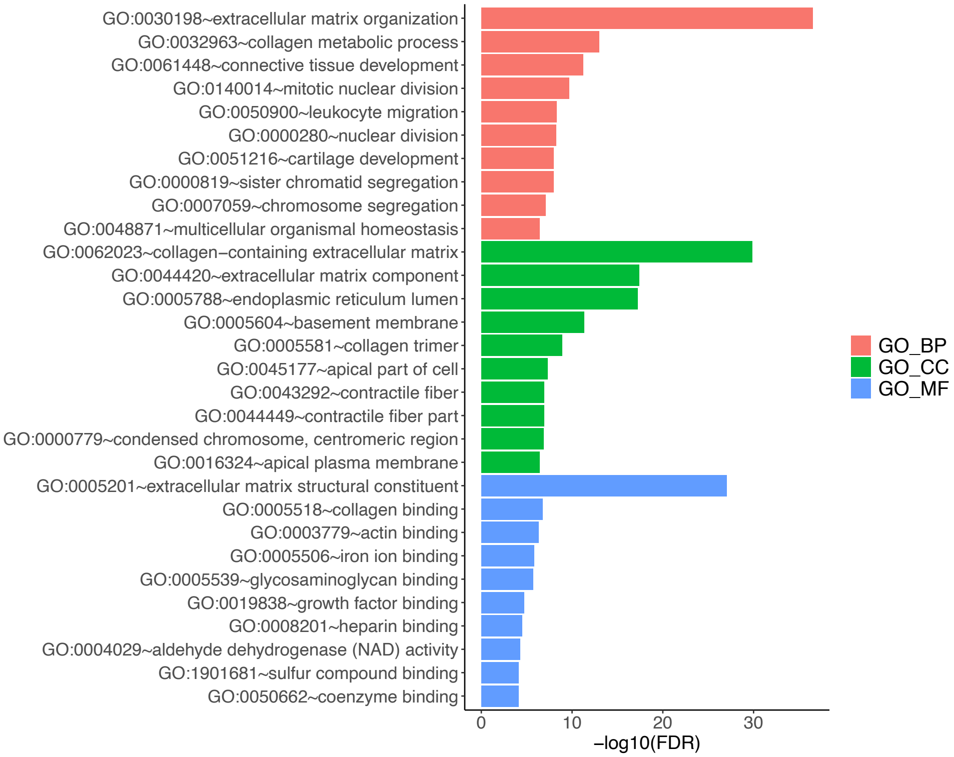

GO:0030198~extracellular matrix organization
GO:0032963~collagen metabolic process
GO:0061448~connective tissue development
GO:0140014~mitotic nuclear division
GO:0050900~leukocyte migration
GO:0000280~nuclear division
GO:0051216~cartilage development
GO:0000819~sister chromatid segregation
GO:0007059~chromosome segregation
GO:0048871~multicellular organismal homeostasis
GO:0062023~collagen−containing extracellular matrix
GO:0044420~extracellular matrix component
GO:0005788~endoplasmic reticulum lumen
GO:0005604~basement membrane
GO:0005581~collagen trimer
GO:0045177~apical part of cell
GO:0043292~contractile fiber
GO:0044449~contractile fiber part
GO:0000779~condensed chromosome, centromeric region
GO:0016324~apical plasma membrane
GO:0005201~extracellular matrix structural constituent
GO:0005518~collagen binding
GO:0003779~actin binding
GO:0005506~iron ion binding
GO:0005539~glycosaminoglycan binding
GO:0019838~growth factor binding
GO:0008201~heparin binding
GO:0004029~aldehyde dehydrogenase (NAD) activity
GO:1901681~sulfur compound binding
GO:0050662~coenzyme binding
0
10
20
30
−log10(FDR)
GO_BP
GO_CC
GO_MF

(D) LIHC

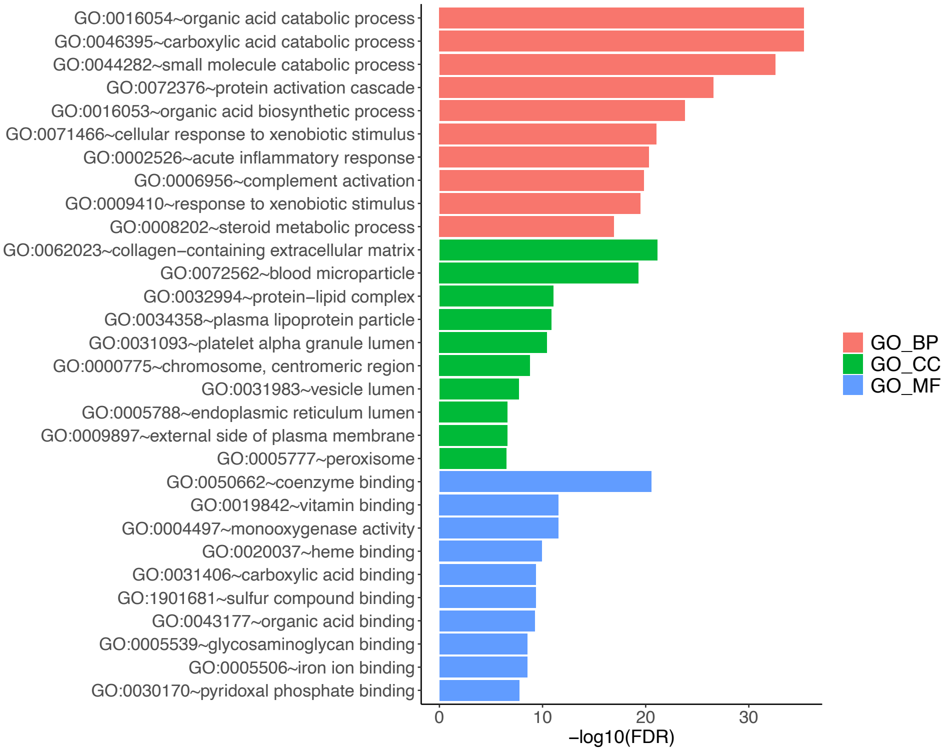
GO:0016054~organic acid catabolic process
GO:0046395~carboxylic acid catabolic process
GO:0044282~small molecule catabolic process
GO:0072376~protein activation cascade
GO:0016053~organic acid biosynthetic process
GO:0071466~cellular response to xenobiotic stimulus
GO:0002526~acute inflammatory response
GO:0006956~complement activation
GO:0009410~response to xenobiotic stimulus
GO:0008202~steroid metabolic process
GO:0062023~collagen−containing extracellular matrix
GO:0072562~blood microparticle
GO:0032994~protein−lipid complex
GO:0034358~plasma lipoprotein particle
GO:0031093~platelet alpha granule lumen
GO:0000775~chromosome, centromeric region
GO:0031983~vesicle lumen
GO:0005788~endoplasmic reticulum lumen
GO:0009897~external side of plasma membrane
GO:0005777~peroxisome
GO:0050662~coenzyme binding
GO:0019842~vitamin binding
GO:0004497~monooxygenase activity
GO:0020037~heme binding
GO:0031406~carboxylic acid binding
GO:1901681~sulfur compound binding
GO:0043177~organic acid binding
GO:0005539~glycosaminoglycan binding
GO:0005506~iron ion binding
GO:0030170~pyridoxal phosphate binding
0
10
20
30
−log10(FDR)
GO_BP
GO_CC
GO_MF

(E) READ

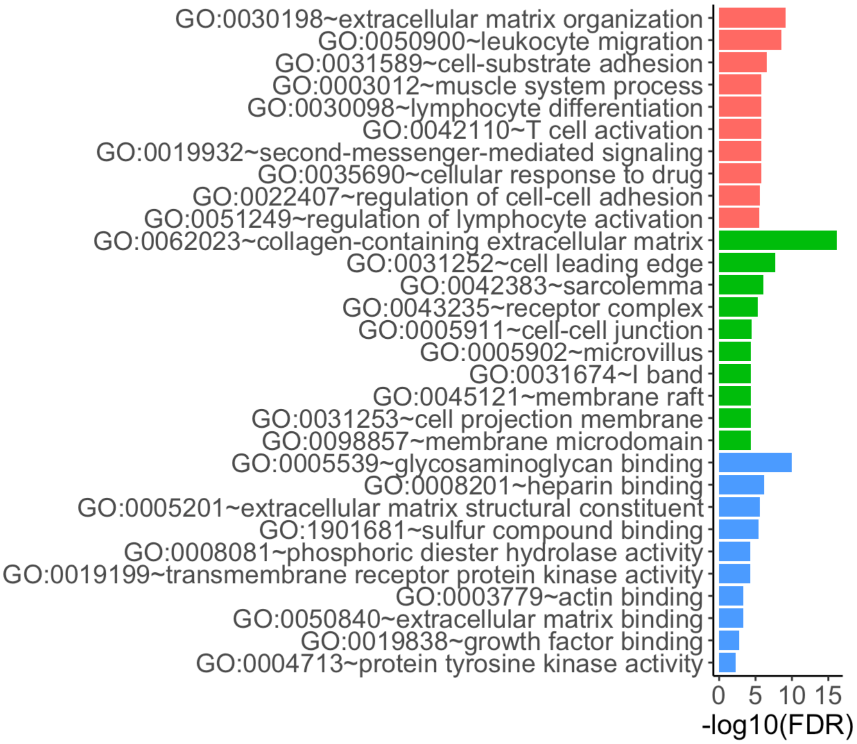
GO:0030198~extracellular matrix organization
GO:0050900~leukocyte migration
GO:0031589~cell-substrate adhesion
GO:0003012~muscle system process
GO:0030098~lymphocyte differentiation
GO:0042110~T cell activation
GO:0019932~second-messenger-mediated signaling
GO:0035690~cellular response to drug
GO:0022407~regulation of cell-cell adhesion
GO:0051249~regulation of lymphocyte activation
GO:0062023~collagen-containing extracellular matrix
GO:0031252~cell leading edge
GO:0042383~sarcolemma
GO:0043235~receptor complex
GO:0005911~cell-cell junction
GO:0005902~microvillus
GO:0031674~I band
GO:0045121~membrane raft
GO:0031253~cell projection membrane
GO:0098857~membrane microdomain
GO:0005539~glycosaminoglycan binding
GO:0008201~heparin binding
GO:0005201~extracellular matrix structural constituent
GO:1901681~sulfur compound binding
GO:0008081~phosphoric diester hydrolase activity
GO:0019199~transmembrane receptor protein kinase activity
GO:0003779~actin binding
GO:0050840~extracellular matrix binding
GO:0019838~growth factor binding
GO:0004713~protein tyrosine kinase activity
0
5
10
15
-log10(FDR)

(F) STAD

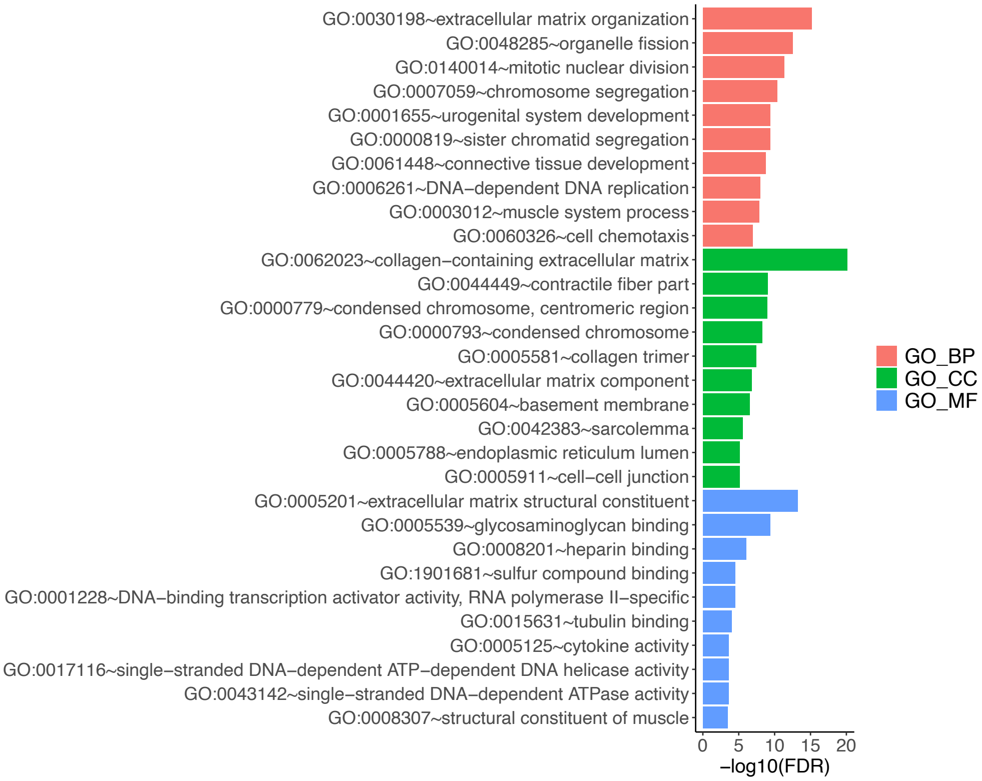
GO:0030198~extracellular matrix organization
GO:0048285~organelle fission
GO:0140014~mitotic nuclear division
GO:0007059~chromosome segregation
GO:0001655~urogenital system development
GO:0000819~sister chromatid segregation
GO:0061448~connective tissue development
GO:0006261~DNA–dependent DNA replication
GO:0003012~muscle system process
GO:0060326~cell chemotaxis
GO:0062023~collagen–containing extracellular matrix
GO:0044449~contractile fiber part
GO:0000779~condensed chromosome, centromeric region
GO:0000793~condensed chromosome
GO:0005581~collagen trimer
GO:0044420~extracellular matrix component
GO:0005604~basement membrane
GO:0042383~sarcolemma
GO:0005788~endoplasmic reticulum lumen
GO:0005911~cell–cell junction
GO:0005201~extracellular matrix structural constituent
GO:0005539~glycosaminoglycan binding
GO:0008201~heparin binding
GO:1901681~sulfur compound binding
GO:0001228~DNA–binding transcription activator activity, RNA polymerase II–specific
GO:0015631~tubulin binding
GO:0005125~cytokine activity
GO:0017116~single–stranded DNA–dependent ATP–dependent DNA helicase activity
GO:0043142~single–stranded DNA–dependent ATPase activity
GO:0008307~structural constituent of muscle
0
5
10
15
20
–log10(FDR)
GO_BP
GO_CC
GO_MF
