# Supplementary material for: LncRNA–miRNA–mRNA Networks of Gastrointestinal Cancers Representing Common and Specific LncRNAs and mRNAs
Source: Front Genet. 2022 Jan 24;12:791919. doi: 10.3389/fgene.2021.791919 (PMC8819090; doi:10.3389/fgene.2021.791919)
Supplement: Supplementary file 4 [file Image2.pdf]

Supplementary Figure 2. OS analysis of genes in patients with TCGA cancers. A: HNSC, B: LIHC, C: READ, D: STAD, E: COAD.

(A) HNSC

KDELC1

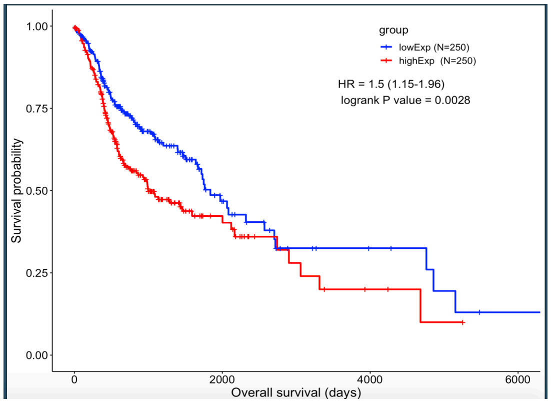

MEIS1

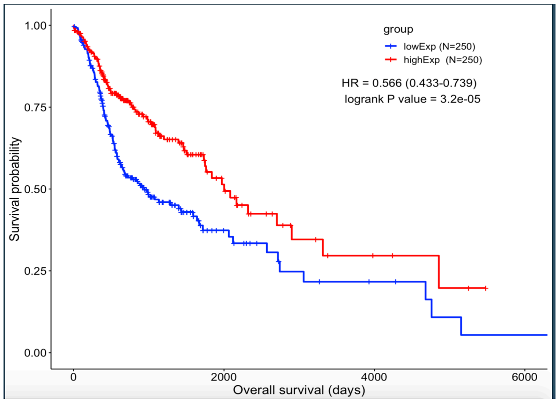

GNA12

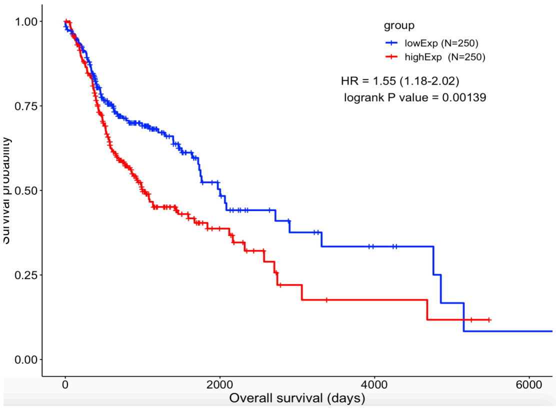

ITGA5

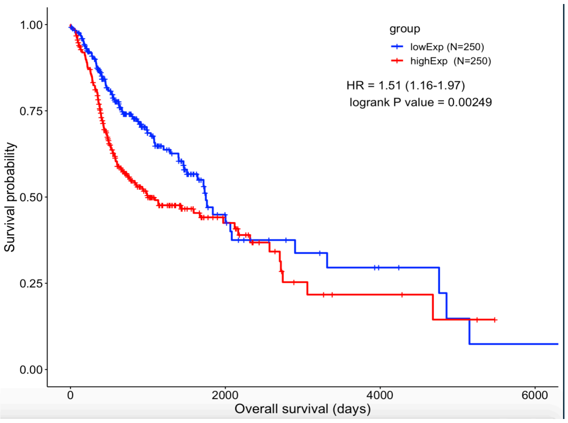

CDCA4

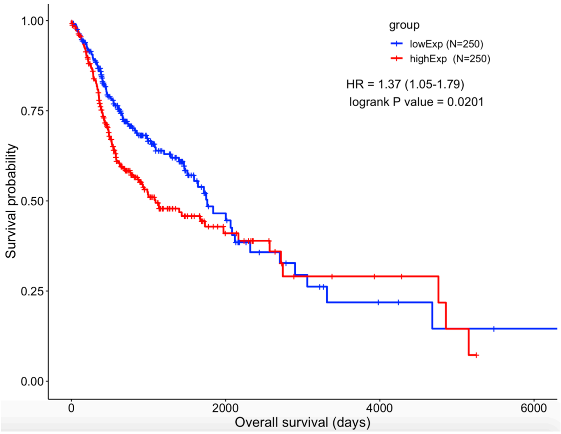

(B) LIHC

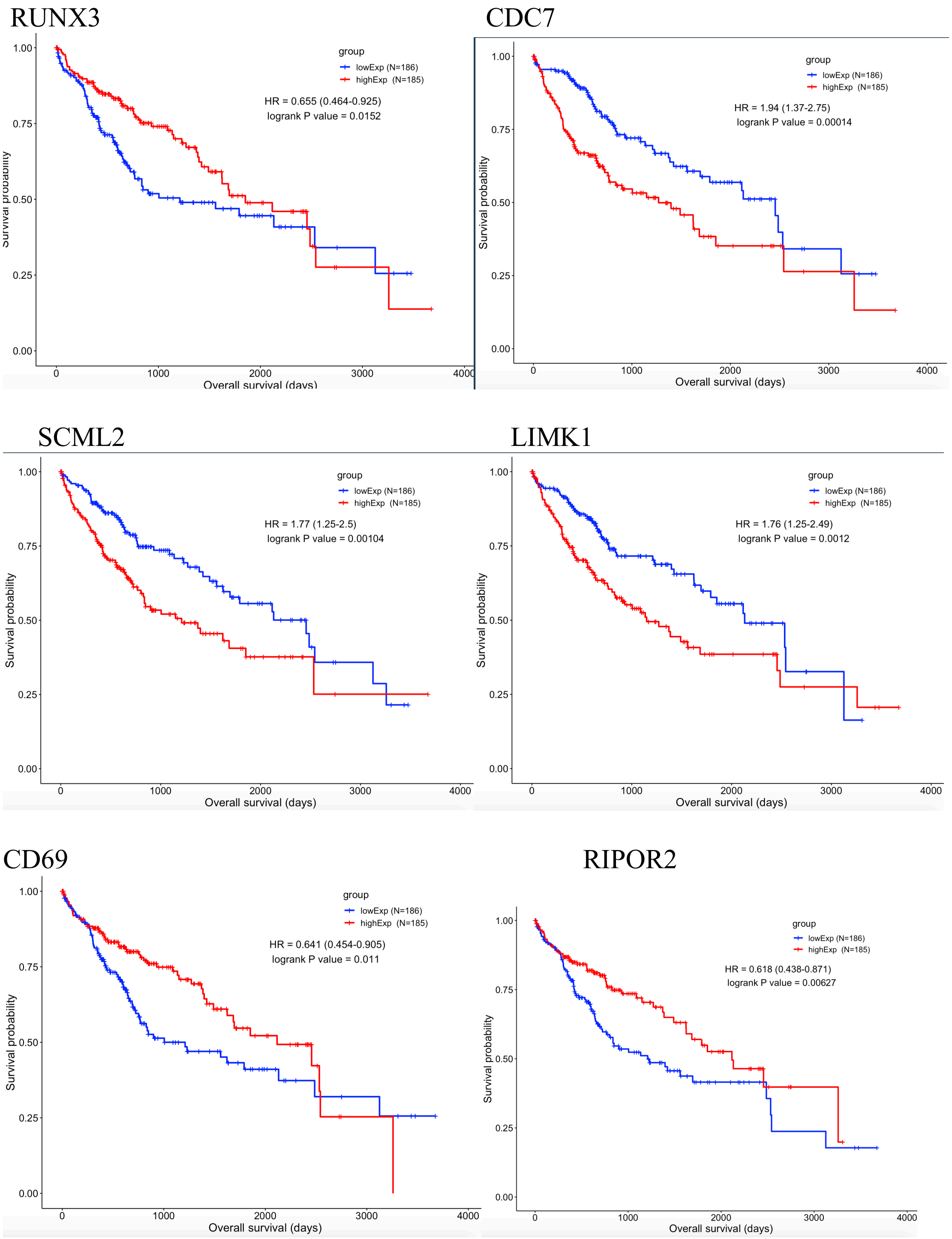
RUNX3
group
lowExp (N=186)
highExp (N=185)
HR = 0.655 (0.464-0.925)
logrank P value = 0.0152
CDC7
group
lowExp (N=186)
highExp (N=185)
HR = 1.94 (1.37-2.75)
logrank P value = 0.00014
SCML2
group
lowExp (N=186)
highExp (N=185)
HR = 1.77 (1.25-2.5)
logrank P value = 0.00104
LIMK1
group
lowExp (N=186)
highExp (N=185)
HR = 1.76 (1.25-2.49)
logrank P value = 0.0012
CD69
group
lowExp (N=186)
highExp (N=185)
HR = 0.641 (0.454-0.905)
logrank P value = 0.011
RIPOR2
group
lowExp (N=186)
highExp (N=185)
HR = 0.618 (0.438-0.871)
logrank P value = 0.00627
Survival probability
Overall survival (days)
0.00
0.25
0.50
0.75
1.00
0
1000
2000
3000
4000

FANCE

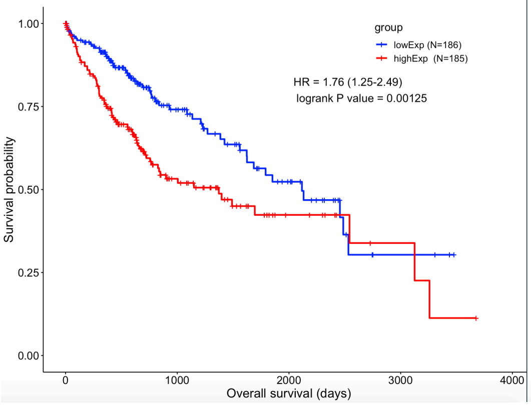

PLXNA1

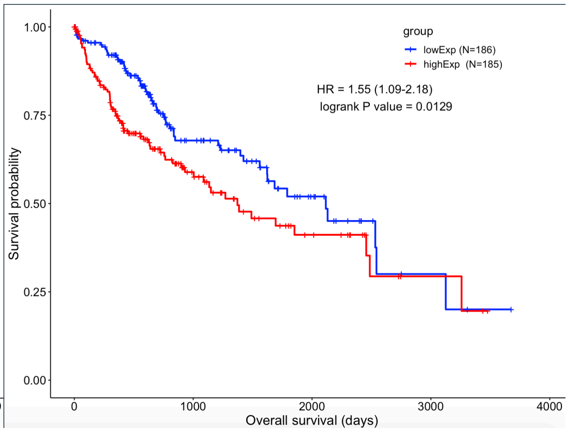

GADD45A

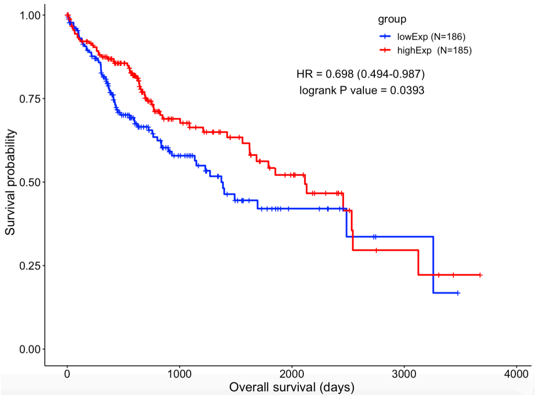

E2F8

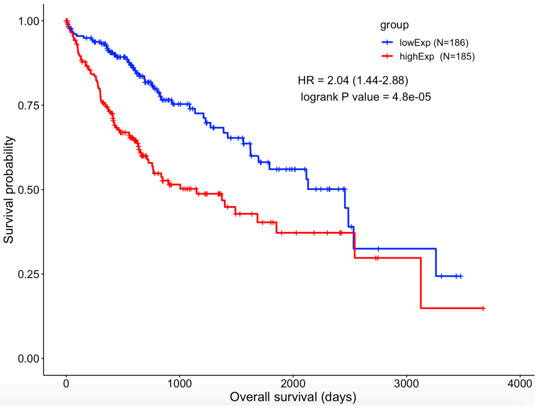

EFNA3

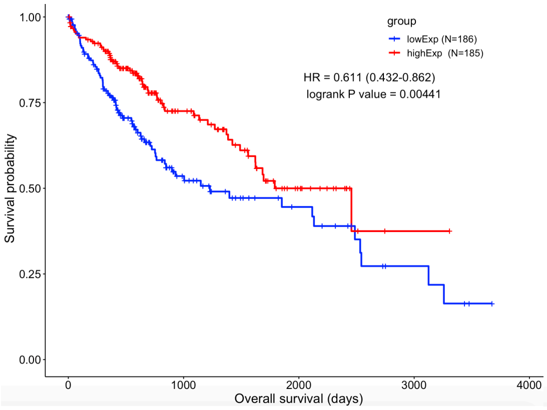

SYBU

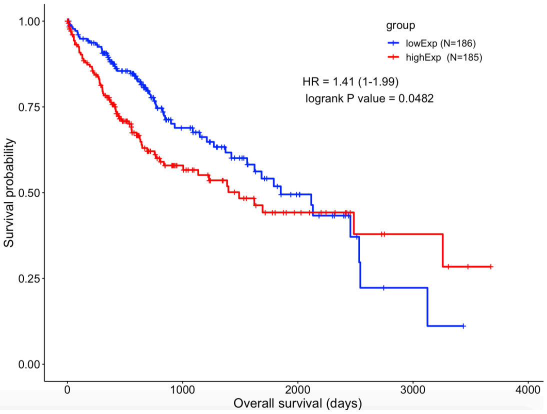

SLC7A11

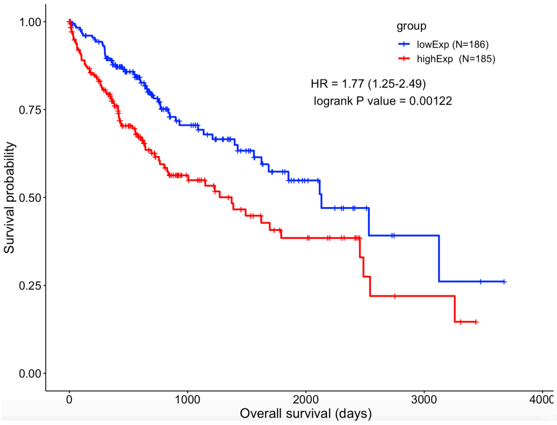

ENAH

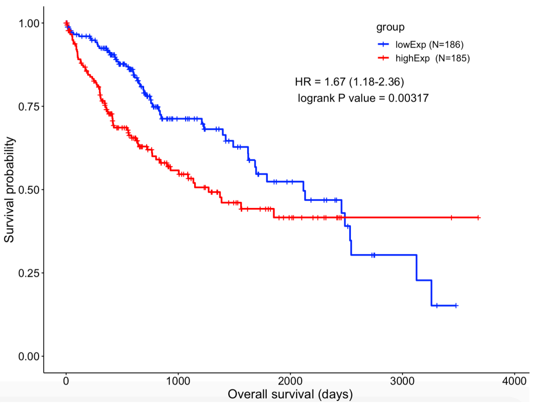

ADAMTSL3

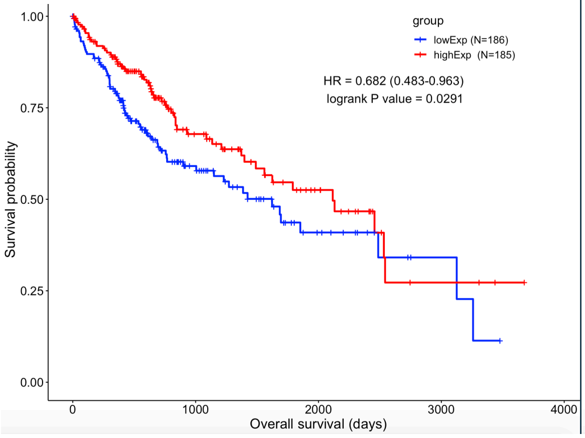

TMEM164

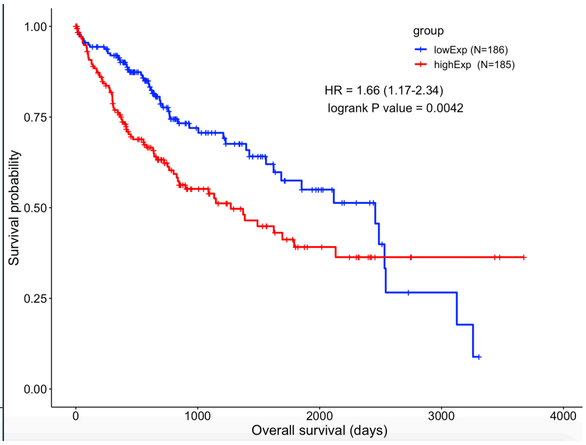

AUTS2

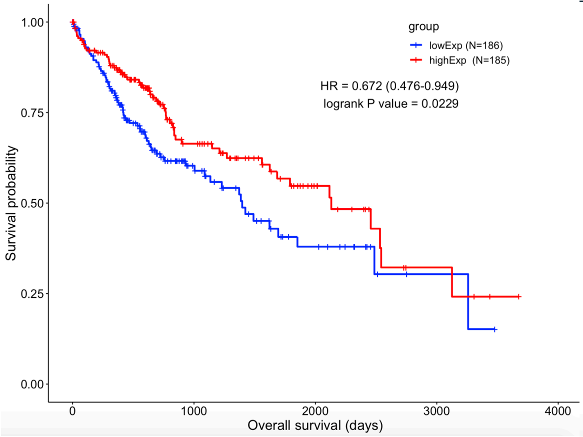

H2AFZ

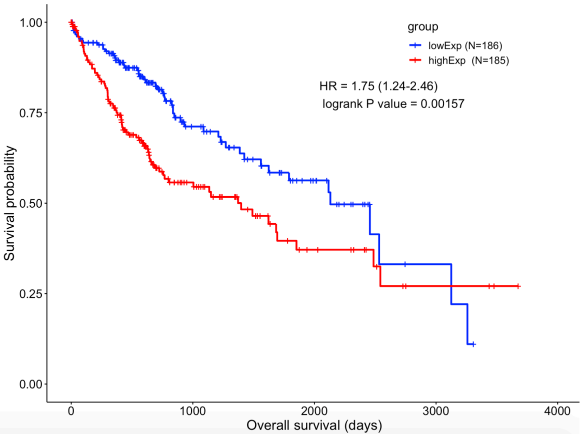

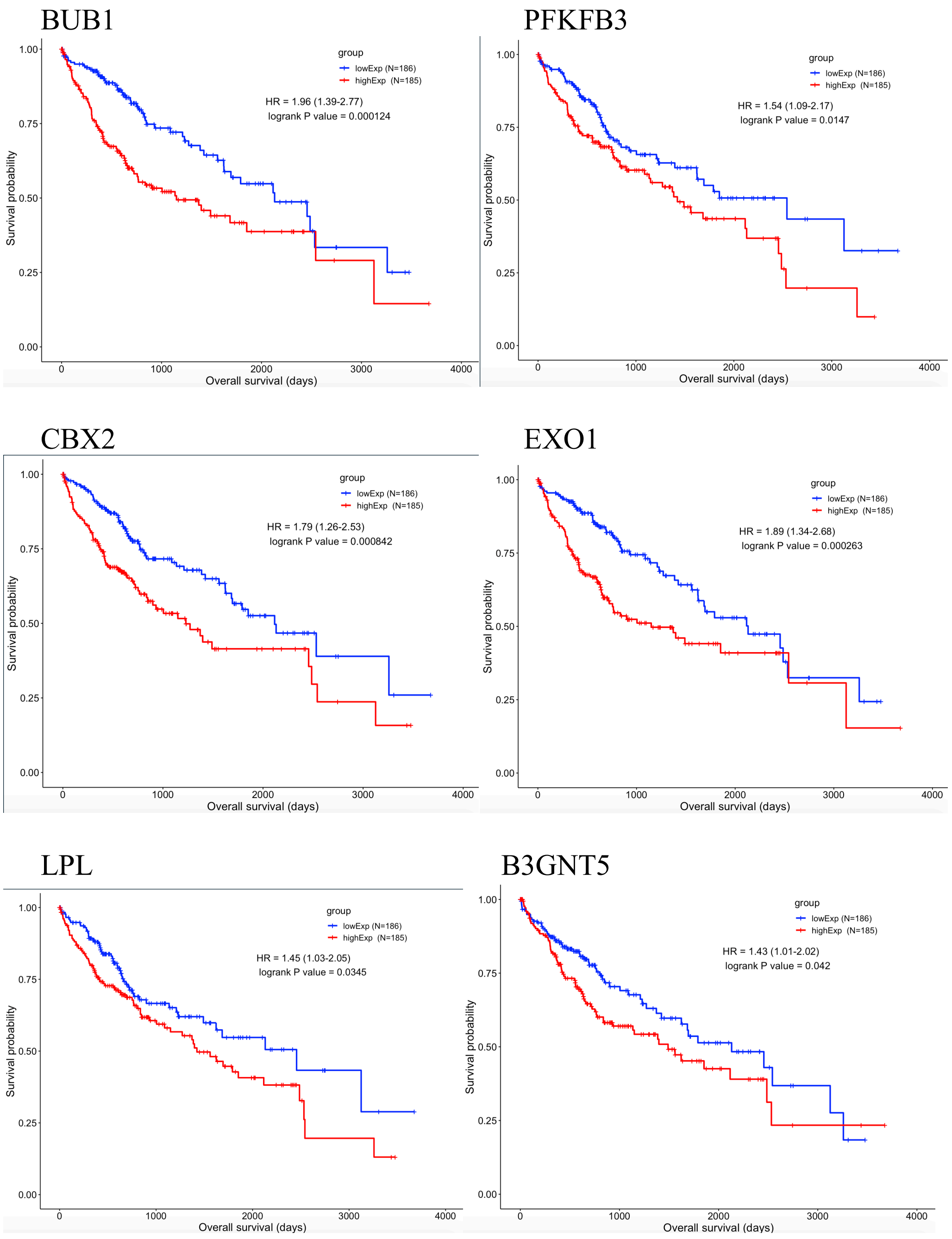
BUB1
group
lowExp (N=186)
highExp (N=185)
HR = 1.96 (1.39-2.77)
logrank P value = 0.000124
Survival probability
Overall survival (days)
PFKFB3
group
lowExp (N=186)
highExp (N=185)
HR = 1.54 (1.09-2.17)
logrank P value = 0.0147
Survival probability
Overall survival (days)
CBX2
group
lowExp (N=186)
highExp (N=185)
HR = 1.79 (1.26-2.53)
logrank P value = 0.000842
Survival probability
Overall survival (days)
EXO1
group
lowExp (N=186)
highExp (N=185)
HR = 1.89 (1.34-2.68)
logrank P value = 0.000263
Survival probability
Overall survival (days)
LPL
group
lowExp (N=186)
highExp (N=185)
HR = 1.45 (1.03-2.05)
logrank P value = 0.0345
Survival probability
Overall survival (days)
B3GNT5
group
lowExp (N=186)
highExp (N=185)
HR = 1.43 (1.01-2.02)
logrank P value = 0.042
Survival probability
Overall survival (days)

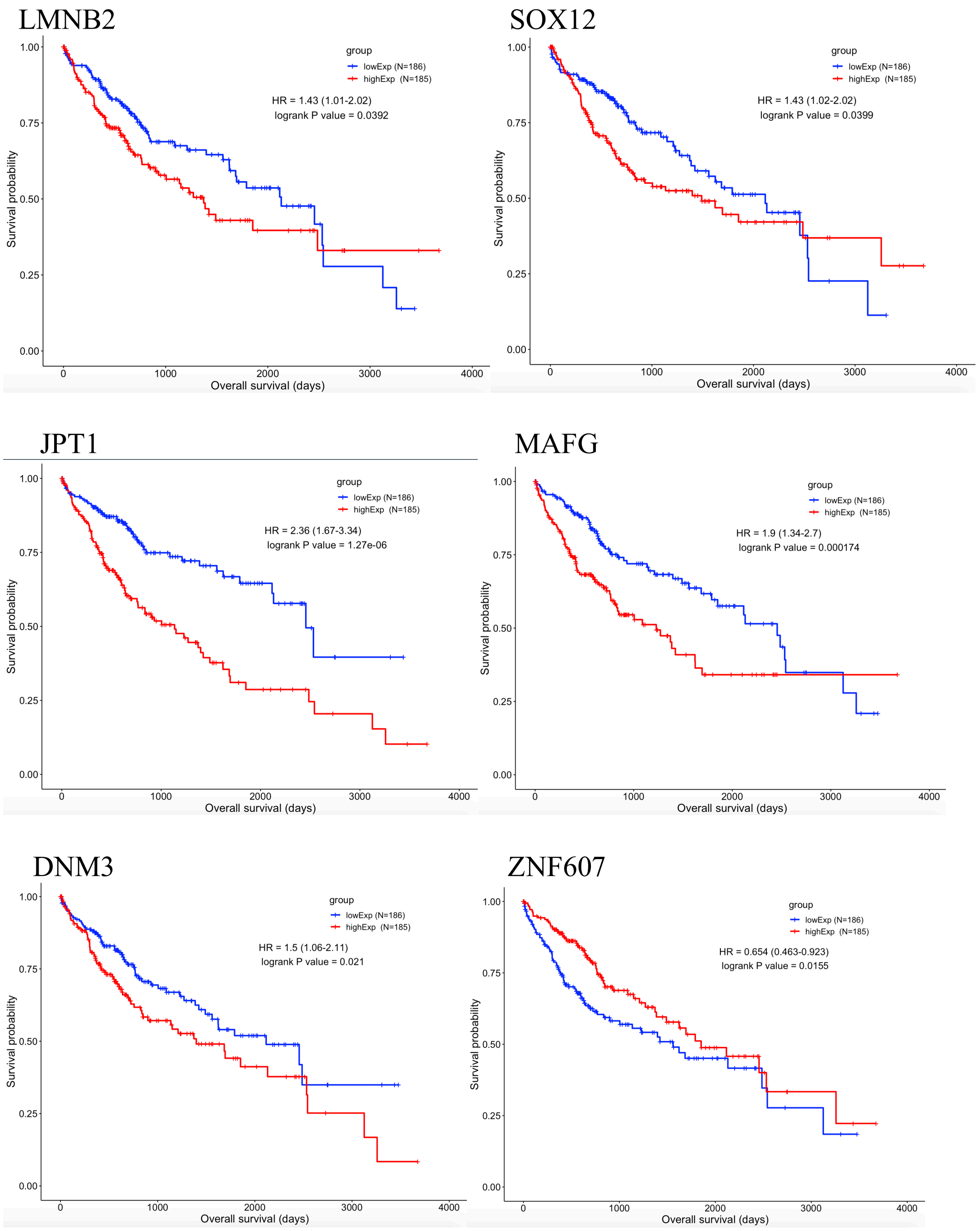

LMNB2
group
lowExp (N=186)
highExp (N=185)
HR = 1.43 (1.01-2.02)
logrank P value = 0.0392
Survival probability
Overall survival (days)
SOX12
group
lowExp (N=186)
highExp (N=185)
HR = 1.43 (1.02-2.02)
logrank P value = 0.0399
Survival probability
Overall survival (days)
JPT1
group
lowExp (N=186)
highExp (N=185)
HR = 2.36 (1.67-3.34)
logrank P value = 1.27e-06
Survival probability
Overall survival (days)
MAFG
group
lowExp (N=186)
highExp (N=185)
HR = 1.9 (1.34-2.7)
logrank P value = 0.000174
Survival probability
Overall survival (days)
DNM3
group
lowExp (N=186)
highExp (N=185)
HR = 1.5 (1.06-2.11)
logrank P value = 0.021
Survival probability
Overall survival (days)
ZNF607
group
lowExp (N=186)
highExp (N=185)
HR = 0.654 (0.463-0.923)
logrank P value = 0.0155
Survival probability
Overall survival (days)

STK39

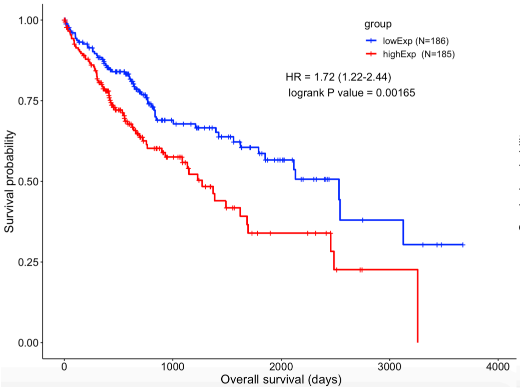

ANKRD13B

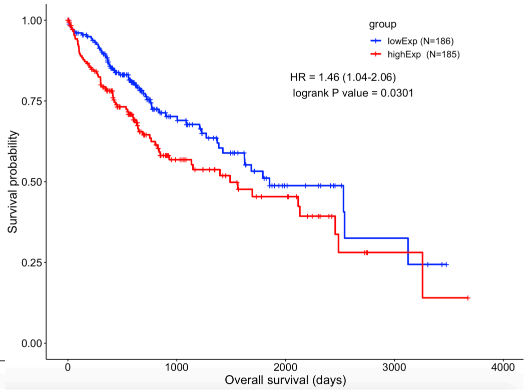

SMOC1

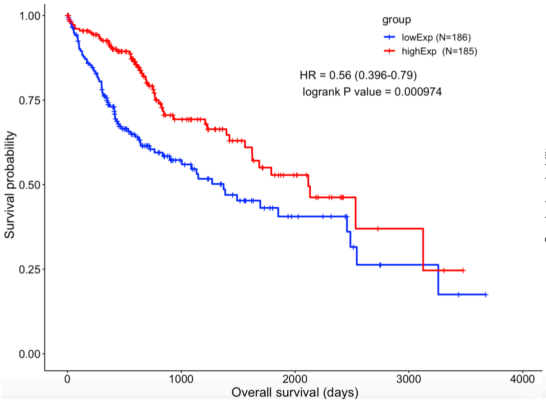

COL15A1

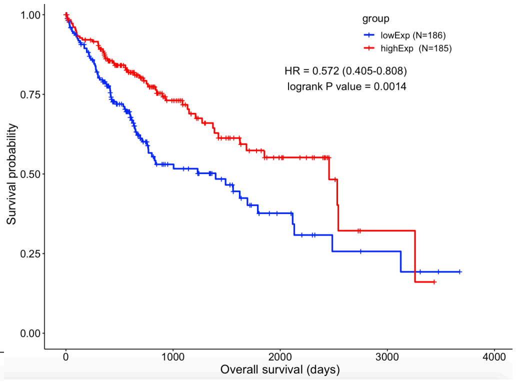

(C) READ

ATP8B2

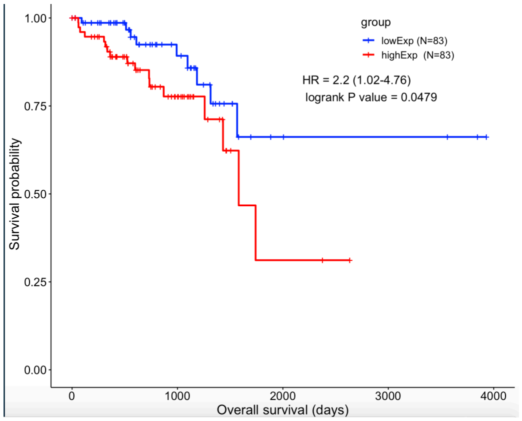

(D) STAD

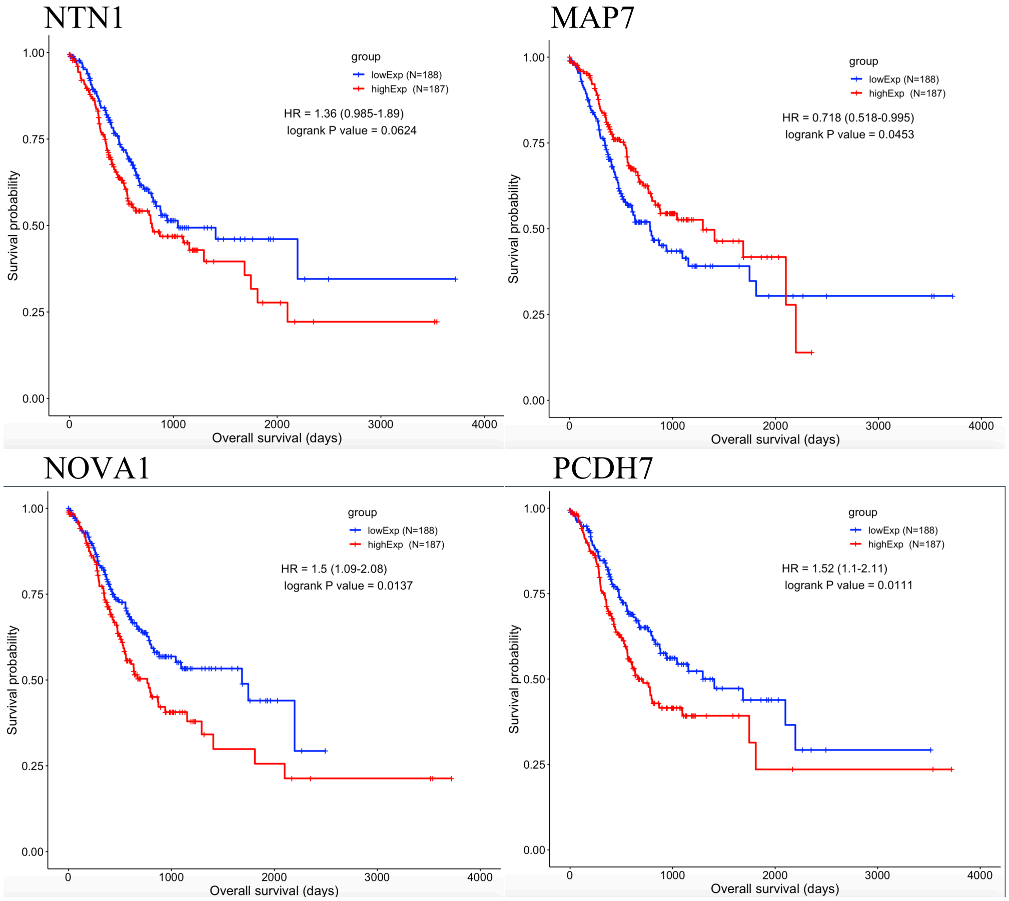

NTN1
group
lowExp (N=188)
highExp (N=187)
HR = 1.36 (0.985-1.89)
logrank P value = 0.0624
MAP7
group
lowExp (N=188)
highExp (N=187)
HR = 0.718 (0.518-0.995)
logrank P value = 0.0453
NOVA1
group
lowExp (N=188)
highExp (N=187)
HR = 1.5 (1.09-2.08)
logrank P value = 0.0137
PCDH7
group
lowExp (N=188)
highExp (N=187)
HR = 1.52 (1.1-2.11)
logrank P value = 0.0111
Survival probability
Overall survival (days)
1.00
0.75
0.50
0.25
0.00
0
1000
2000
3000
4000

(E) COAD

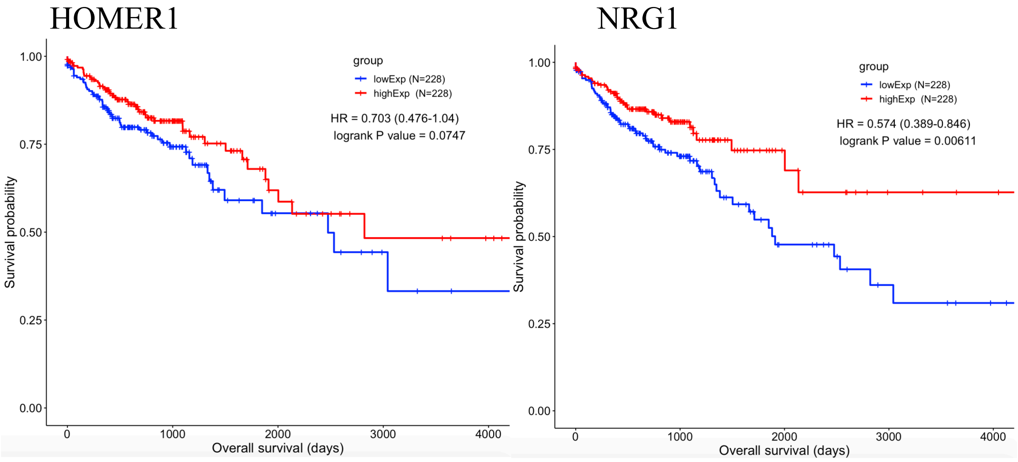

HOMER1
group
lowExp (N=228)
highExp (N=228)
HR = 0.703 (0.476-1.04)
logrank P value = 0.0747
NRG1
group
lowExp (N=228)
highExp (N=228)
HR = 0.574 (0.389-0.846)
logrank P value = 0.00611
Survival probability
Overall survival (days)
1.00
0.75
0.50
0.25
0.00
0
1000
2000
3000
4000
