# Supplementary material for: LncRNA–miRNA–mRNA Networks of Gastrointestinal Cancers Representing Common and Specific LncRNAs and mRNAs
Source: Front Genet. 2022 Jan 24;12:791919. doi: 10.3389/fgene.2021.791919 (PMC8819090; doi:10.3389/fgene.2021.791919)
Supplement: Supplementary file 6 [file Image3.pdf]

**Supplementary Figure 3.** MRNAs with significant altered expression across tumor stages in ceRNA networks of GI cancers.

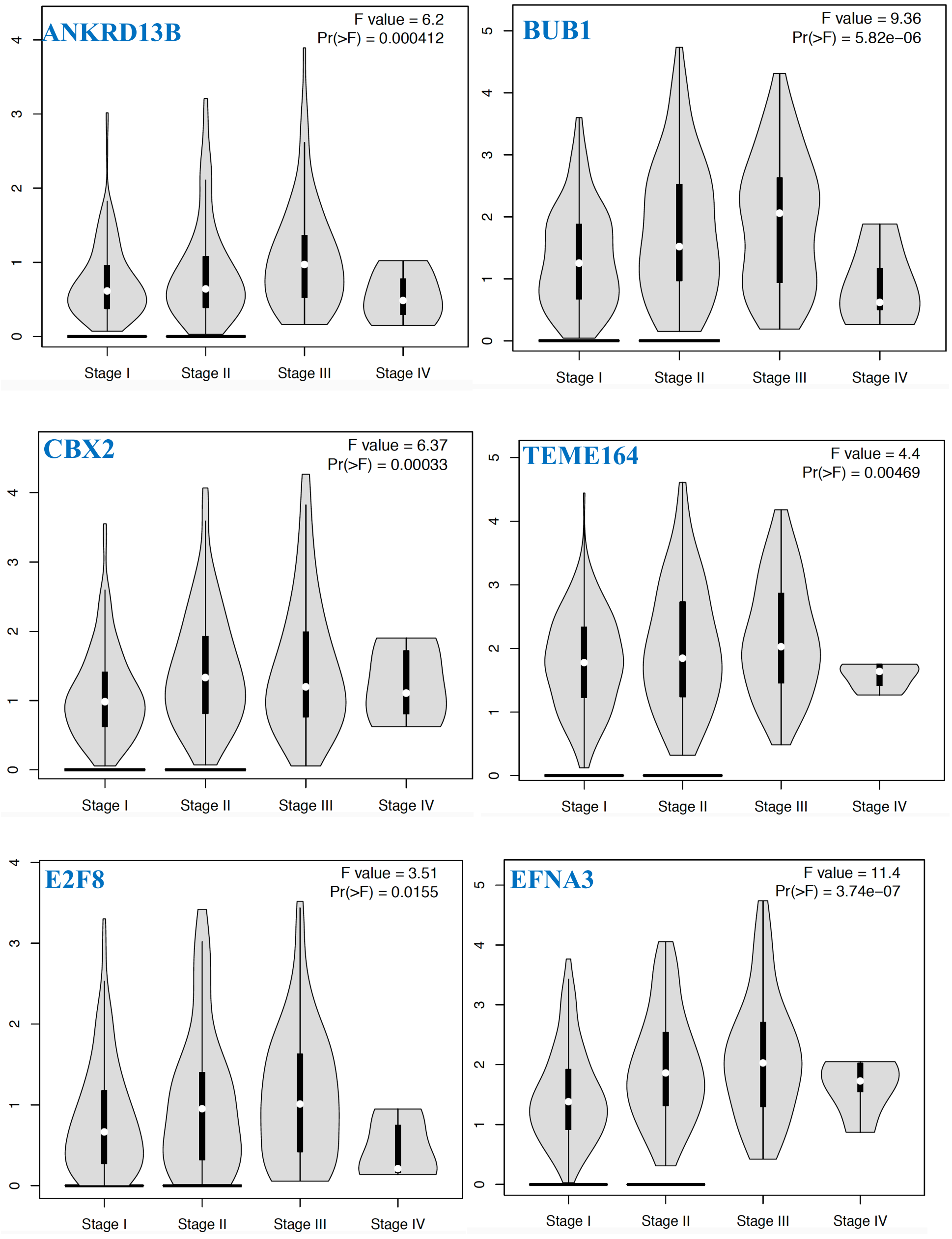

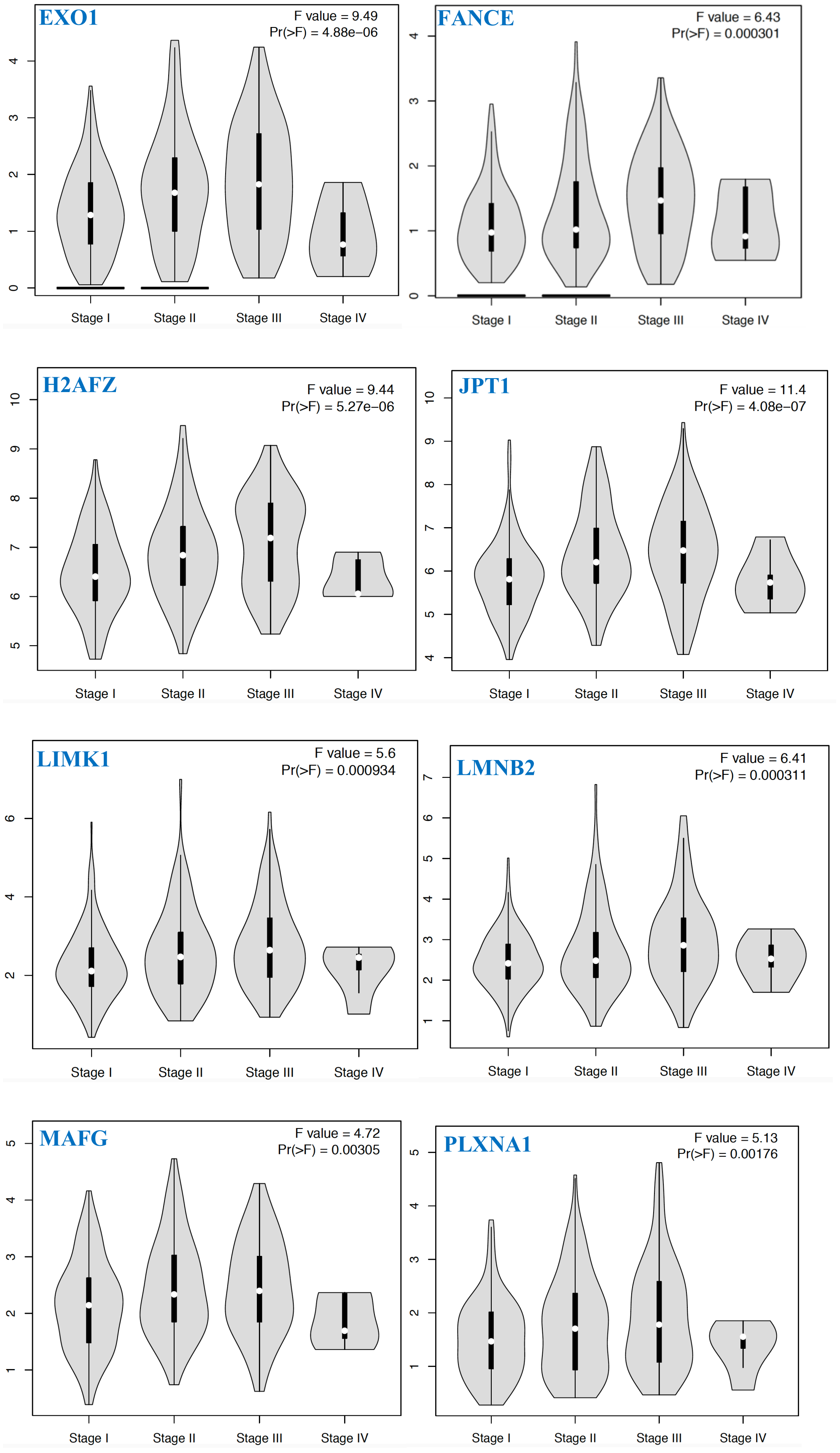
EXO1
F value = 9.49
Pr(>F) = 4.88e−06
FANCE
F value = 6.43
Pr(>F) = 0.000301
H2AFZ
F value = 9.44
Pr(>F) = 5.27e−06
JPT1
F value = 11.4
Pr(>F) = 4.08e−07
LIMK1
F value = 5.6
Pr(>F) = 0.000934
LMNB2
F value = 6.41
Pr(>F) = 0.000311
MAFG
F value = 4.72
Pr(>F) = 0.00305
PLXNA1
F value = 5.13
Pr(>F) = 0.00176
Stage I
Stage II
Stage III
Stage IV

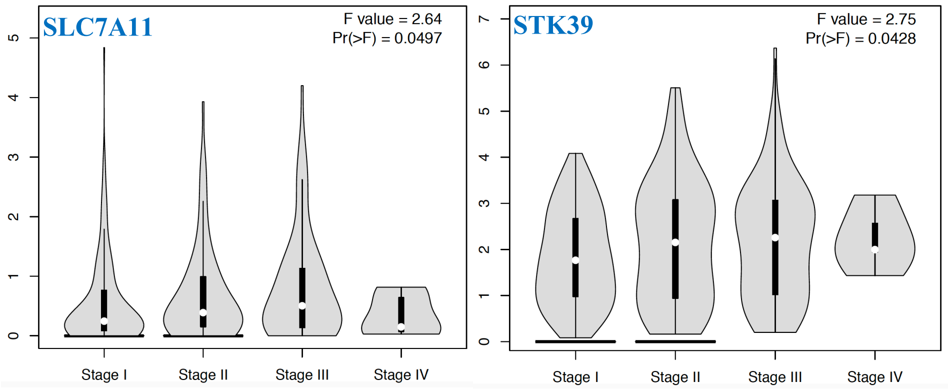
SLC7A11
F value = 2.64
Pr(>F) = 0.0497
Stage I
Stage II
Stage III
Stage IV
STK39
F value = 2.75
Pr(>F) = 0.0428
Stage I
Stage II
Stage III
Stage IV

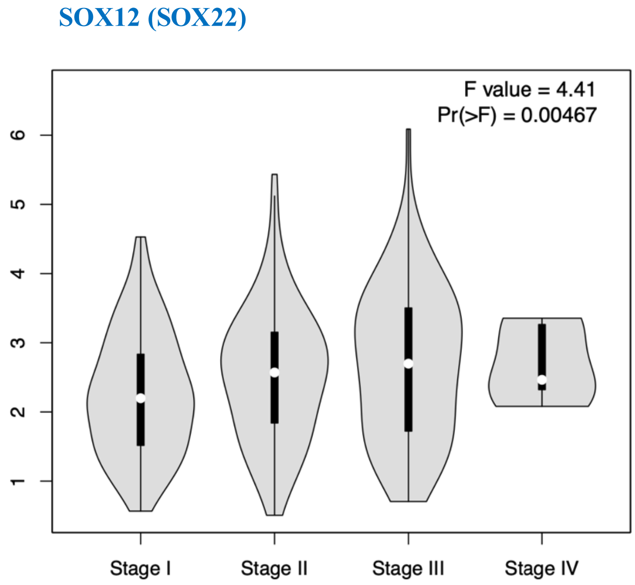
SOX12 (SOX22)
F value = 4.41
Pr(>F) = 0.00467
Stage I
Stage II
Stage III
Stage IV
