# Supplementary figures and images for: LncRNA–miRNA–mRNA Networks of Gastrointestinal Cancers Representing Common and Specific LncRNAs and mRNAs
Source: Front Genet. 2022 Jan 24;12:791919. doi: 10.3389/fgene.2021.791919 (PMC8819090; doi:10.3389/fgene.2021.791919)

**Supplementary Figure 1.** CeRNA networks of LIHC (A), HNSC (B), READ (C), and ESCA (D).

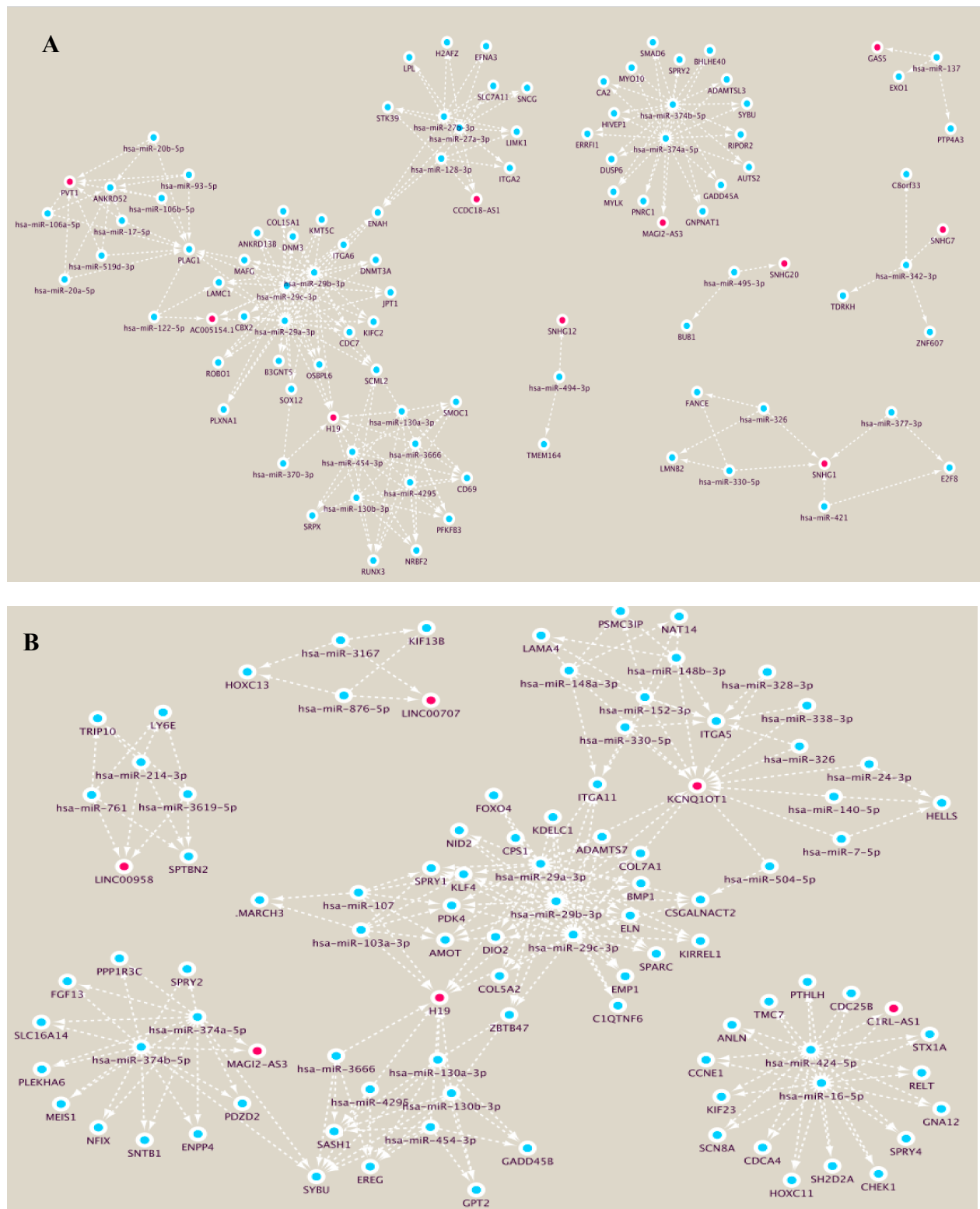

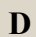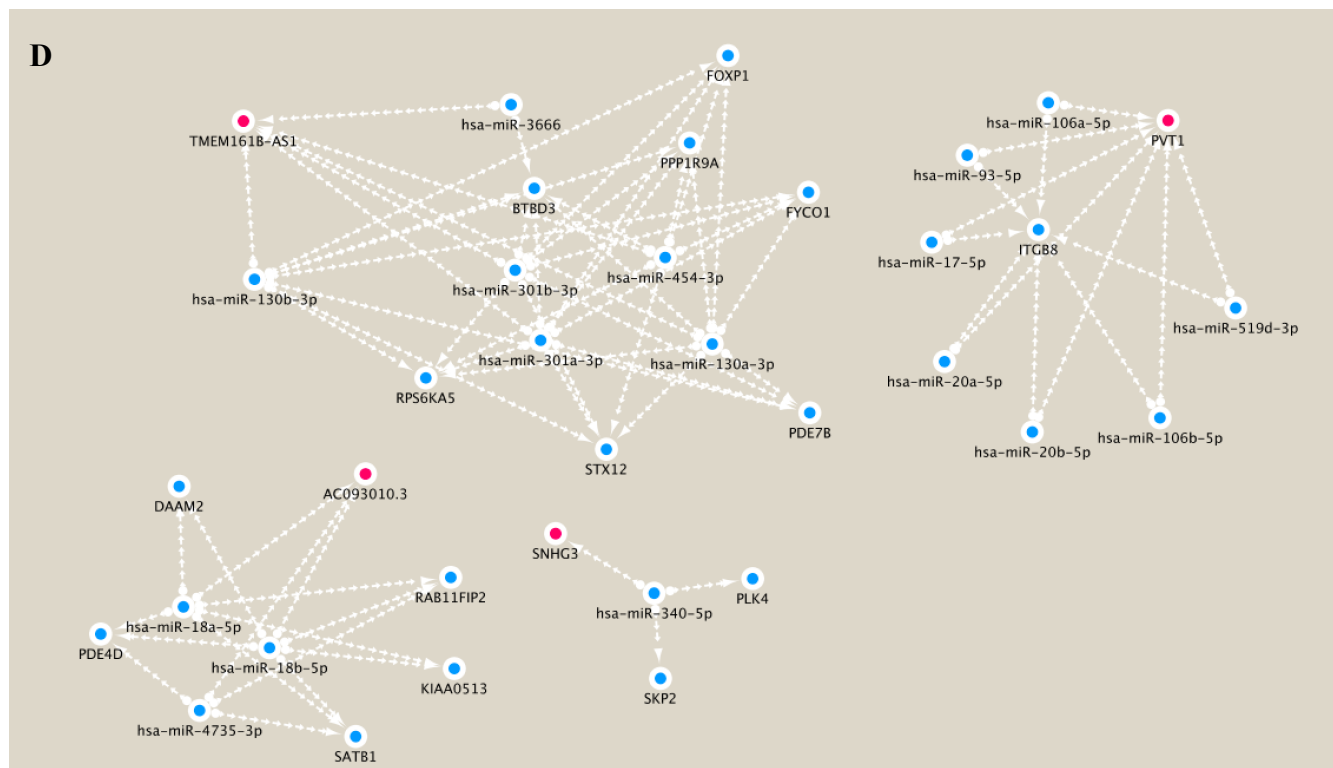

Supplement: Supplementary file 10 [file Image1.pdf]
